# A genome-wide association study of occupational creativity and its relations with well-being and career success

**DOI:** 10.1038/s42003-024-06686-5

**Published:** 2024-09-05

**Authors:** Wen-Dong Li, Xin Zhang, Kaili Yu, Yimo Zhu, Nianyao Du, Zhaoli Song, Qiao Fan

**Affiliations:** 1 Department of Management, CUHK Business School, The Chinese University of Hong Kong, Hong Kong, China; 2 Department of Human Resource Management, School of Business, Shanghai University of Finance and Economics, Shanghai, China; 3 Department of Management and Organization, National University of Singapore, Singapore, Singapore; 4 Department of Statistics and Data Science, National University of Singapore, Singapore, Singapore; 5 Centre for Quantitative Medicine, Duke-NUS Medical School, National University of Singapore, Singapore, Singapore

**Keywords:** Human behaviour, Behavioural genetics

## Abstract

Creativity is one defining characteristic of human species. There have been mixed findings on how creativity relates to well-being, and little is known about its relationship with career success. We conduct a large-scale genome-wide association study to examine the genetic architecture of occupational creativity, and its genetic correlations with well-being and career success. The SNP-*h*^*2*^ estimates range from 0.08 (for managerial creativity) to 0.22 (for artistic creativity). We record positive genetic correlations between occupational creativity with autism, and positive traits and well-being variables (e.g., physical height, and low levels of neuroticism, BMI, and non-cancer illness). While creativity share positive genetic overlaps with indicators of high career success (i.e., income, occupational status, and job satisfaction), it also has a positive genetic correlation with age at first birth and a negative genetic correlation with number of children, indicating creativity-related genes may reduce reproductive success.

## Abstract (web summary)

A GWAS study reveals that genes related to occupational creativity are associated with both positive and negative well-being variables, positively correlate with career success, and negatively correlate with reproductive success

## Introduction

Creativity plays a crucial role in shaping not only the optimal functioning of individuals, but also the development and well-being of human societies and civilizations^1–3^. A significant amount of research endeavors has thus been devoted to identifying what factors contribute to creativity. Among these endeavors, individual characteristics have received ample research attention in the literature, which perhaps dates back to Galton’s landmark research on the heredity of creative genius^4^. Indeed, twin studies have shown sizable genetic influences on creativity^5–7^. Recent molecular genetics research has further revealed specific genetic variants that may be responsible for the heritability of creativity^8,9^.

Genetic research on creativity has shed light on the notion that creativity is perhaps one of the few defining characteristics of the human species^10^, the importance of which looms large in the new era of artificial intelligence^11^. Yet, this line of inquiry is not without limitations. First, affected by the proposition that creativity is domain specific^12,13^, the prior research on adult creativity has primarily concentrated on either specific occupations (e.g., artists or scientists) or a narrow group of people with unique creative characteristics with small samples^14^. This has limited the scope of creativity research, because creativity matters for a broader spectrum of work and occupations beyond artists and scientists^13^. Second, the lack of a more inclusive and comprehensive approach to creativity has also hampered the investigation of the genetic architecture of creativity using large samples that allow us to tackle important questions in the field, such as whether and how the genetic architecture of creativity overlaps with that of health and well-being variables. The stereotype that there may be a positive correlation between creativity and mental disorder— perhaps dating back to Aristotle^15^—suggests a possible negative phenotypic correlation between creativity and well-being^16–19^. A recent meta-analysis, however, imposes a challenge to this stereotype by reporting a small, but significant positive phenotypic correlation (.14) between creativity and well-being^20^. Probing genetic correlations between creativity and well-being may contribute to a more nuanced understanding of the genetic etiology and the nature of such relationships^21^. Third, another stereotype of creative people is that they often struggle with their careers^22,23^. This has in fact been supported by census data^24,25^ showing that artists often earn less income—an objective indicator of career success^26^—than counterparts in other occupations, suggesting a negative phenotypic correlation between creativity and career success. Research has also demonstrated that artists experienced higher levels of job satisfaction—a subjective indicator of career success—than incumbents in other occupations^27^, which suggests a positive phenotypic relationship between creativity and career success. Thus, a large-scale study on the nature and genetic etiology of the relationship between creativity and a relatively more comprehensive spectrum of career success variables (e.g., income, occupation status, job satisfaction, and reproductive success) may shed light on the inconsistent findings on the phenotypic relationship between creativity and career success^21^.

We thus conducted a large-scale genome-wide association study using data from the U.K. biobank (*N* = 219,473) and three independent replication samples (*N* = 26,975) to examine the above three questions. The objective of this research is three-fold. First, we draw from the literatures on creativity and organizational research^28^ and developed two more inclusive and comprehensive creativity measures to capture creativity in a broader fashion. *Creativity* is often defined as the generation of ideas, products, problem solutions, approaches and practices that are both novel and useful^1,3,29^. Creativity is a multifaceted construct and has been shown to be very difficult to assess^13,30^. Prior research on adult creativity has primarily concentrated on artistic and scientific creativity^14^ and thus neglected another important form of creativity related to novel and useful management practices and approaches in organizations, which we call managerial creativity (e.g., the introduction of modern assembly line)^31^. Indeed, the literature in organizational research has highlighted that managerial jobs, particularly those at senior and leadership levels, require job incumbents to come up with novel and appropriate ideas and practices to initiate changes in the workplace and to deal with new challenges in the ever-changing business world^28,32–34^. Such novel challenges loom large in today’s business world wherein extant approaches to manage customers’ needs, supply chains, and employee motivation and turnover are constantly interrupted and modified by broader political, economic, and technological changes^34,35^. Accordingly, with our first measure of *occupational creativity*, we distinguished the three types of creativity (artistic, scientific, and managerial) as they are reflected in the three categories of occupations based on the U.K. Standard Occupational Classification (SOC) 2000 systems (we also combined the three creative occupations to general a categorial variable of overall occupational creativity). Furthermore, in order to provide a common metric to assess creativity across various occupations (because job incumbents in occupations beyond the three creative occupations may also exhibit high levels of creativity), we capitalized on the literature on occupational classification/analyses and constructed an omnibus measure of creativity from the U.S. Department of Labor’s Occupational Information Network (O*NET)^36^ by a crosswalk linking the U.K. SOC system to the O*NET SOC system^37^. We adopted three items from O*NET (e.g., tapping into participants’ abilities to generate novel ideas and thinking creatively) to access occupational *creative achievement* as a continuous variable. Employing the two major measures of creativity enables us to further explore possible genetic polymorphisms at the whole genome level in our GWAS. To further examine the distinction among the three different forms of creativity, we also examine their different genetic underpinnings and how they are distinctively related to personal traits (e.g., intelligence, educational achievement, personality, and physical height) at the whole genome level (also for well-being and career success variables).

Second, we investigated the genetic correlations between occupational creativity and a number of health and well-being variables including bipolar disorder and schizophrenia —traditionally theorized and found as positive correlates with creativity^8,9,38–41^—and others (e.g., subjective well-being, BMI, autism, longevity, cannabis misuse, and alcohol use).

Third, we examined the genetic correlations between occupational creativity with a wide range of important indicators of career success, including income, occupation status, job satisfaction, and reproductive success (number of children and age at first birth). We also compared such genetic correlations among the three different forms of creativity. We further examined the genetic correlations between creativity and well-being and career success variables after partialling out genetic influences associated with intelligence, an important cognitive predictor of creativity (3), as well as educational achievement. Overall, our investigation contributes to the scholarship on creativity by expanding its scope with including artistic, scientific, managerial creativity and a more general metric of creative achievement, examining the genetic architecture of occupational creativity in a large-scale study, and probing the genetic correlations between occupational creativity with well-being and career success.

## Results

### Phenotypical measures of occupational creativity

Due to its multifaceted nature, we assessed creativity with two measures (five variables) based on the major job responsibilities of participants’ occupations (Table S1). The first was a categorial measure capturing three types of occupational creativity: artistic, scientific, and managerial, as manifested in occupations that require generating novel and useful ideas (in total four binary variables). The literature has primarily focused on two forms of creativity: artistic and scientific^14^. It has overlooked another form of creativity in managerial and leadership jobs—managerial creativity, the importance of which looms large when organizations face ever-changing business environments to deal with novel challenges related to supply-chain, customer needs, and employee management^34,35^. We thus further included a third category: managerial creativity. We captured the three types of occupational creativity via categorizing participants’ occupations based on the U.K. SOC 2000 system into creative occupations (artistic, scientific, and managerial) according to the major responsibilities of the occupations that require generating new and appropriate ideas (Method). In addition to treating the three types of creative occupations (artistic, scientific, and managerial occupations versus conventional/noncreative occupations) as three binary variables, we also combined them to generate a variable of overall occupational creativity (i.e., 1 = either of the three creative occupations, 0 = conventional occupations). Thus the first categorical measure yielded four binary creativity variables: artistic, scientific, managerial, and overall. In order to assess occupational creativity from a broader scope beyond the three types of occupational creativity with a common metric across various types of occupations, our second measure of creativity gauged creative achievement using three items from O*NET by linking the U.K. SOC 2000 occupation codes in the U.K. Biobank (UKB) data to the occupation codes from O*NET^37^.

In our GWAS analyses with the UKB discovery data, we included 125,803 participants of European ancestry with 67,848 classified as holding creative occupations that have high levels of occupational creativity, and 57,955 as holding conventional (less creative) occupations (Table 1, S2, S3 & Figure S1). Among participants with creative occupations, 6,625 were artists, 18,225 scientists, and 42,998 managers. In addition, 219,473 participants with available data on the creative achievement phenotype were included in the discovery stage. The average creative achievement score was 3.24 ranging from 1.03 to 4.74.

**Table 1:** Summary of the creativity phenotypes in the UKB discovery sample

	*N*	Case	Control	Genomic control λ
Artistic creativity	64,580	6625	57955	1.147
Scientific creativity	76,180	18225	57955	1.147
Managerial creativity	100,953	42998	57955	1.096
Overall occupational creativity	125,803	67848	57955	1.147
	N	Mean	SD	Genomic control λ
Creative Achievement	219,473	3.24	0.79	1.310

In our replication analyses, we included three independent datasets: the UKB follow-up dataset (*N* = 23,249), Add Health Wave IV dataset (*N* = 1,461), and Wisconsin Longitudinal Study (WLS; *N* = 2,265). The mean age (years) of participants was 59.8 in the UKB follow-up dataset (32.72% male), 37.8 in the Add Health dataset (37.60% males), and 68.4 in the WLS cohort (35.79% males; Table S5).

### GWAS for occupational creativity

**Fig. 1:**
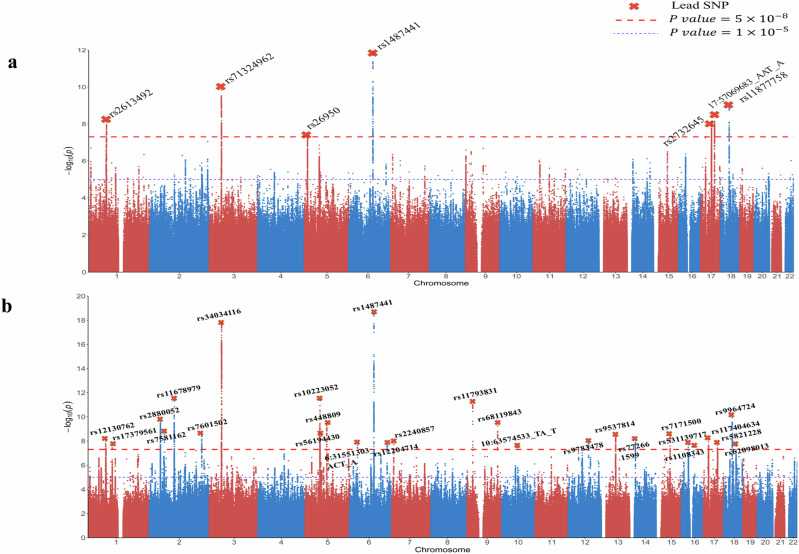
Manhattan plot of GWAS analysis for creativity traits in the UKB discovery sample. Results are shown for **a** overall occupational creativity (*n* = 125,803), and **b** creative achievement (*n* = 219,473). The y axis represents -log_10_(*p*-value) for association with each phenotype, and the x axis represents genomic position based on human genome build 37. The cross in red represents independent genome-wide significant association signals, labelled by names of gene or nearest genes.

We conducted GWAS analyses on 9,804,641 variants that passed quality control (QC) with a minor allele frequency (MAF) of more than 1% for the five creativity variables in UKB discovery data (see method and ref)^42^. The linkage disequilibrium (LD) score regression intercept, which ranged from 1.004 to 1.049, indicated the expected polygenicity for these traits^43^ (see Table S6 and Fig. 1 and S2 for Manhattan plots).

For GWAS performed in this study, a locus was defined by an index lead SNP (*P* < 5 × 10^−8^) with its flanking 500-kb region in either direction. Nineteen loci were identified to be significantly linked to one of these five creativity variables at the *P* < 1.0 × 10^−8^, with P-value accounted for multiple testing correction for five traits. Six were shared by at least two traits (see Tables S7–1 and Figures S3 and S4 for QQ plots). Additionally, 12 more loci displayed conventional genome-wide significance for either of these five creativity variables, with a *P*-value of < 5.0 × 10^−8^ (see Tables S7–1). Altogether 31 loci were carried out for the replication.

We subsequently evaluated associations of these top loci with the creativity variables in the three independent replication cohorts (see above). All analyses were adjusted for age, sex and main principal components. For a total of 43 variants at 31 top loci identified in UKB discovery, 28 variants at 19 loci showed genome-wide significant for different traits at 1.0 × 10^−8^ in the meta-analysis (see Tables S7–1). Three proxy variants (*r*^2^ > 0.9 with the lead variant) were selected since the lead variant was not presented in the meta-analysis results. Tables S7–2 summarizes findings of our meta-analyses based on both the discovery and replication samples. No heterogeneity was observed for the effect size across cohorts for these replicated variants (*P* ≥ 0.0737, see Tables S7–2**)**.

Among the 19 top loci, five were identified as significant for overall occupational creativity, one for artistic creativity at chromosome 18 gene *CELF4*, one for managerial creativity and scientific creativity at chromosome 6 *MIR2113/PNKY*, and three for scientific creativity (chromosome 1 *NEGR1*, chromosome 3 *GPX1/USP4/NICN1*, chromosome 5 *NDUFAF2/PART1/ZSWIM6*). Several top loci identified for creativity traits were overlapped with the genes previously reported for psychiatric traits such as schizophrenia (*ZSWIM6*)^40^, bipolar disorder (micro RNA *MIR2113*)^41^, neuroticism (*CELF4*)^44^, and autism (*NEGR1*)^45^.

The identified top loci harbor genes involved in various neuronal functions. For example, gene *NEGR1* encodes protein neuronal growth regulator 1 (*Negr1*) of 46KDa. Negr1 is highly expressed in the cerebral cortex, hippocampus, and cerebellum in the brain, and is a member of the immunoglobulin LON family^46^. It accumulates in the neurotransmitter GABAergic inhibitory synapses of neurons^47^. As neurotransmitters play a crucial role in creativity^48^, the mutation of NEGR1 may have implications for the trait of creativity. Another gene *CELF4* (*CUGBP*, ELAV-like family 4) is related to a neural RNA-binding protein, predominantly expressed in the central nervous system. *CELF4* plays a critical role in various neuronal functions and development, particularly in RNA processing^49^.

Sex-specific analyses were also conducted, which identified nine novel loci for either males and females (see Table S8). Among these, one novel signal was found for the creative achievement variable at gene *SALL1* on chromosome 16 in males, while two novel signals were identified at gene *NSUN3* on chromosome 3 and gene *MAPKAP1* on chromosome 9 in females (also see Figures S5 for Manhattan plots).

To test whether the aggregate estimates of genetic effects are associated with creativity phenotypes, we constructed polygenic scores (PGS) based on the GWAS summary statistics in the independent UKB follow-up dataset, Add health, and WLS data utilizing C + T (clumping + thresholding)^50^ and PRS-SC approaches^51^ (Table S18). The results from both approaches are similar. The PGSs were significantly associated with all 5 creativity phenotypes in the UK follow-up dataset (model fitting P for PGS ≤ 6.53 × 10^−5^). PGS accounted for a small amount of variance of leadership position, with an incremental R2 up to 1.34%, on top of age, sex and top PCs. For Addhealth and WLS, due to the small sample and heterogeneity of the sample, the PGSs were not significant, except for scientific creativity, and creative achievement in WLS sample (*p* ≤ 6.77 × 10^−5^). The findings of small predictive power were generally similar to those reported previously in social sciences^20^.

### Heritability and genetic correlations among the creativity variables across sex

**Fig. 2:**
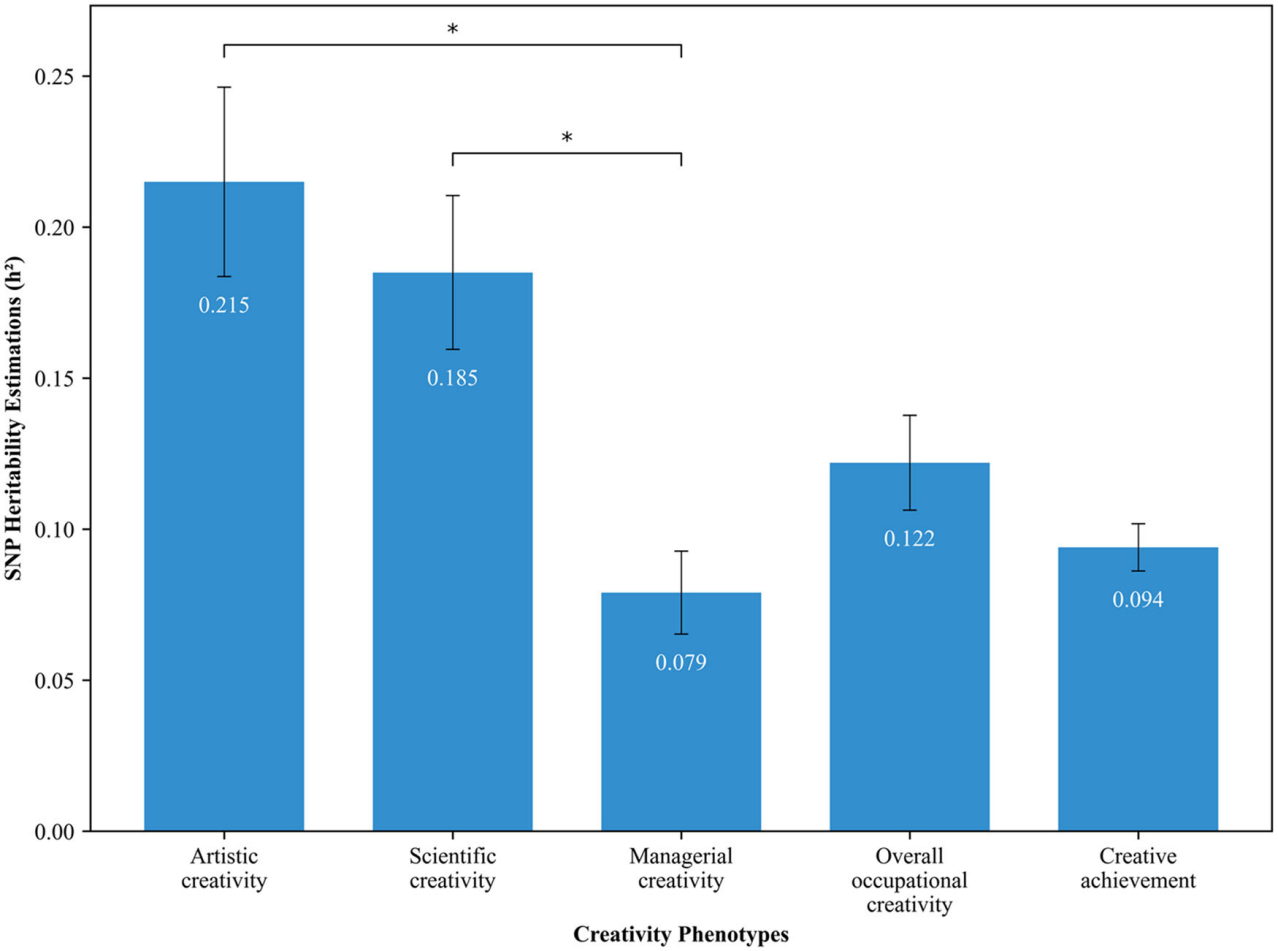
Summary of common SNP heritability estimations for creativity phenotypes from GWAS results in UKB data. *Liability-scale h*^*2*^ for artistic creativity, scientific creativity, managerial creativity, and overall occupational creativity, *Observed-scale h*^*2*^ for creative achievement. Vertical bars represent 95% CIs. Asterisks denote the comparison of heritability between artistic creativity, scientific creativity, and managerial creativity at Bonferroni-adjusted p-values < 0.01667.

We calculated common SNP heritability (SNP-*h*^*2*^) for the five creativity variables in the UKB discovery sample (Table S9**and** Fig. 2). Using GWAS summary statistics, we applied LD-score regression to estimate the proportion of variance in liability to creativity traits that was explained by the aggregated effect of the SNPs^52^. The SNP-*h*^*2*^ estimates were 0.22 (95% CI [0.18, 0.25]), 0.18 (95% CI [0.16, 0.21]). 0.08 (95% CI [0.06, 0.09]) and 0.12 (95% CI [0.11, 0.14]), for artistic, scientific, managerial and overall occupational creativity. For creative achievement, the SNP-*h*^*2*^ estimate was 0.0939 (95% CI [0.09, 0.10]). The SNP-*h*^*2*^ estimates for artistic and scientific creativity were significantly higher than that for managerial creativity. The SNP-*h*^*2*^ estimates observed in this study were smaller than the heritability estimates reported in twin studies^5–7^. This is probably related to the fact that creativity is a multifaceted construct and thus it is likely to be affected by thousands of genetic variants (i.e., polygenity). Yet, the SNP-*h*^*2*^ estimates are similar to those reported for variables studied in social sciences, including personality traits and subjective well-being^21^.

The five creativity traits had moderate to strong genetic correlations among them (Table 2). These findings suggest a possibly similar significant genetic basis for the different types of creativity variables, and that certain types of creativity may be more closely related than others. In addition, there were high genetic correlations of creative phenotypes between males and females (Table S10).

**Table 2:** Genetic correlations for creativity phenotypes in the UKB sample

Overall Genetic Correlation (S.E.)	Artistic creativity	Scientific creativity	Managerial creativity	Overall occupational creativity
Scientific creativity	0.7691 (0.0403)			
Managerial creativity	0.6395 (0.0506)	0.6603 (0.0344)		
Overall occupational creativity	0.8205 (0.031)	0.8809 (0.0148)	0.9271 (0.0081)	
Creative achievement	0.8235 (0.028)	0.9235 (0.0156)	0.8347 (0.0223)	0.9608 (0.0108)

### Genetic correlations between occupational creativity and personal traits

We then examined the genetic overlaps between creativity variables and personality traits, well-being, and success variables using summary statistics from previous GWAS research (See Methods; Table S11). In order to correct for multiple testing, we set the significance level at a false discovery rate (FDR) < 0.05^53^. With respect to personal traits, we included intelligence, educational achievement, the big five personality traits (openness, neuroticism, extraversion, agreeableness, and conscientiousness), risk tolerance and physical height. The creativity literature^3,14,29,30,32^ has theorized and found significant relationships between creativity with intelligence and personality traits (e.g., openness, risk tolerance, and extraversion). Physical height has been shown to affect one’s career success and has also been factored in important indicators of physical health^54^.

**Fig. 3:**
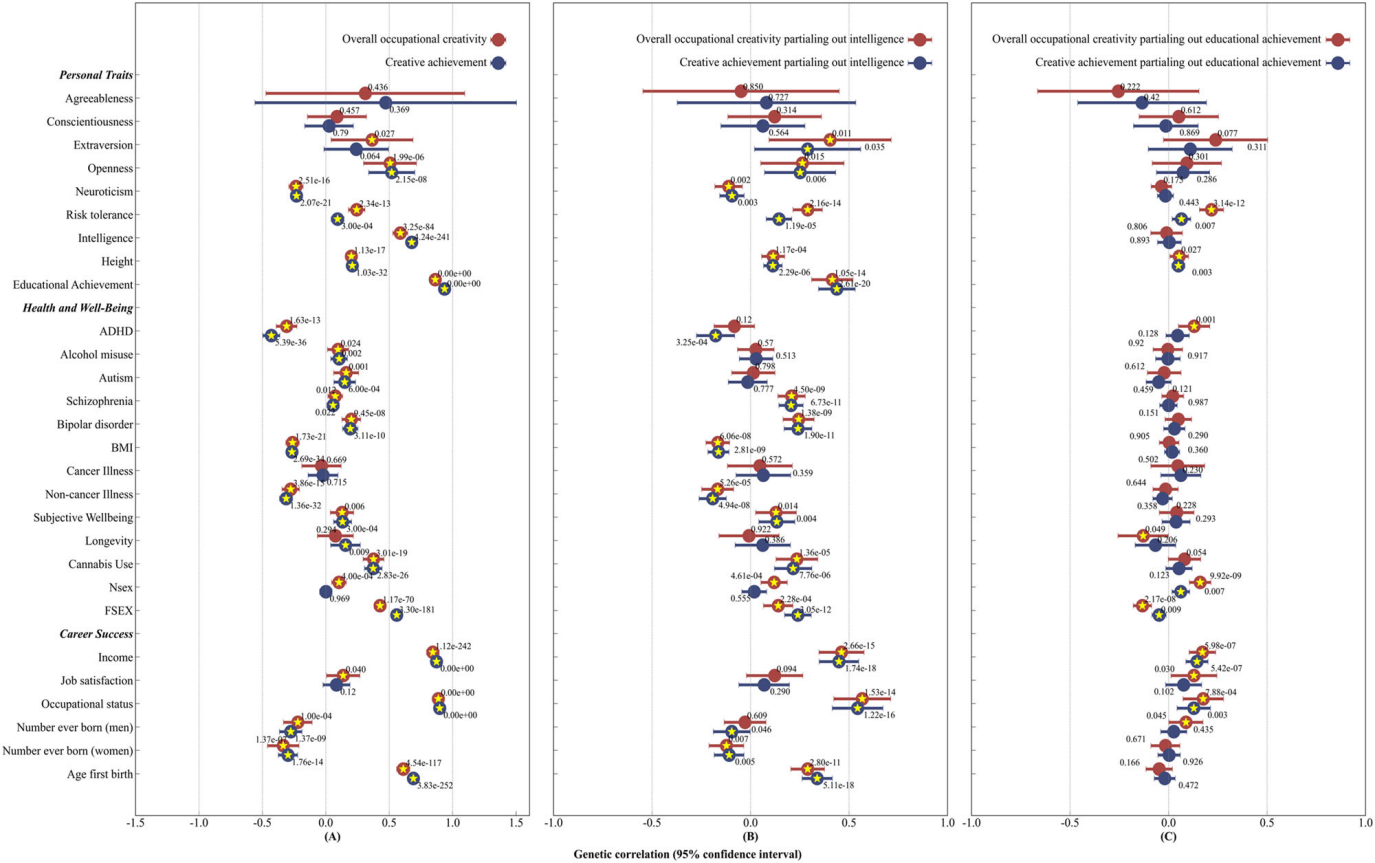
Genetic correlations of creativity phenotypes with personality traits, health and well-being, and career success. **A** Overall occupational creativity and creative achievement before partialling out genetic variance related to intelligence and educational achievement. **B** Overall occupational creativity and creative achievement after partialling out genetic variance related to intelligence. **C** Overall occupational creativity and creative achievement after partialling out genetic variance related to educational achievement. *P* values for the genetic correlations are reported above each dot. Horizontal bars represent 95% CIs. Yellow asterisks denote the genetic correlations at FDR < 0.05.

Our analyses (Fig. 3 and Table S12) revealed significant and relatively large genetic correlations between the two major creativity variables (overall occupational creativity combining all the three types of creativity and creative achievement) and intelligence, as well as educational achievement. The two creativity variables also shared significant and moderate to large genetic overlaps with openness. We also recorded findings of moderate genetic correlations of creativity with neuroticism and height.

**Fig. 4:**
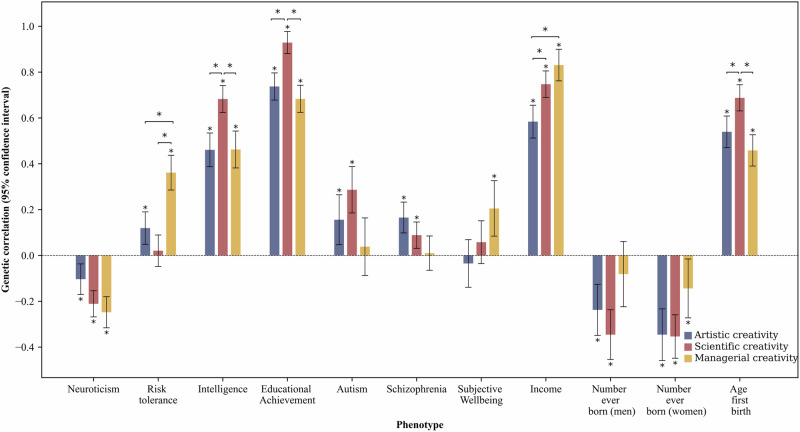
Genetic correlations of artistic, scientific, and managerial creativity with outcomes. Vertical bars represent 95% CIs. The error bars are imposed on R^2^. Asterisks denote the genetic correlations at FDR < 0.05 and the comparison of genetic correlations at Bonferroni-adjusted p-values < 0.00152.

We observed significant differences in genetic correlations across the three different types of occupational creativity (Tables S12 and S13; Fig. 4). Scientific creativity (*r*_*g*_ = 0.68, 95% CI = [0.62, 0.74]) had a larger genetic correlation with intelligence than artistic creativity (*r*_*g*_ = 0.46, 95% CI = [0.39, 0.53]; for comparison: *P* = 3.72 × 10^−6^) and managerial creativity (*r*_*g*_ = 0.46, 95% CI = [0.38, 0.54]; *P* = 1.43 × 10^−5^). Managerial creativity had a larger genetic correlation with risk tolerance than artistic creativity and scientific creativity. Artistic creativity had a lower genetic correlation (in absolute value) with neuroticism than managerial creativity.

### Genetic correlations between occupational creativity and well-being

We selected well-being variables that are either phenotypically or genetically related to creativity as reported previously in examining their genetic correlations with occupational creativity (Fig. 3 and Table S12). We found positive genetic correlations of creativity with autism—an indicator of low levels of well-being, and with an indicator of high levels of well-being: subjective well-being. Creativity also had negative genetic correlations with indicators of low well-being: BMI and non-cancer illness.

Similar to previous research, creativity was also genetically and positively correlated with bipolar disorder, schizophrenia, alcohol misuse, cannabis use, and age at first sextual intercourse. It also had a significant and negative genetic correlation with ADHD.

Our analyses also revealed significant differences in such genetic correlations across the three types of occupational creativity (Tables S12 and S13; Fig. 4). For example, artistic creativity had a larger positive genetic correlation with schizophrenia than managerial creativity. Managerial creativity had a larger genetic correlation with subjective well-being than artistic creativity. Scientific creativity had a larger genetic correlation with autism than managerial creativity.

### Genetic correlations between occupational creativity and career success

The relationships of creativity with career success have received little research attention in the literature. We found (Fig. 3 and Table S12) significant and positive genetic correlations between creativity and indicators of high levels of career success including income, and occupational status. The genetic correlation between overall occupational creativity and job satisfaction was significant.

With respect to reproductive success, both indicators of number of children (number of children for men and for women) had negative genetic correlations with creativity. Age of first birth had a positive genetic correlation with creativity.

Regarding the difference in genetic correlations across the three types of occupational creativity, we found (Tables S12 and S13; Fig. 4) that both managerial creativity and scientific creativity had greater positive genetic correlations with income than artistic creativity. Scientific creativity had a larger genetic correlation with age at first birth than both artistic and managerial creativity. The genetic correlations (in absolute value) between number of children and scientific creativity were larger than those with managerial creativity.

### Genetic correlations of occupational creativity with well-being and career success after partialling out genetic influences associated with intelligence and educational achievement

Thus, it is important to partial out genetic influences associated with intelligence or educational achievement when examining the above genetic correlations. Such analyses shed light on the role of non-cognitive factors underlying such genetic correlations. To estimate the genetic correlations after partialling out the genetic variance of intelligence or educational attainment, we used the Genomic Structure Equation Modeling (SEM) (Genomic SEM)^55^. Our results (See Methods; Tables S14 and S15; Fig. 3) show that partialling out genetic variance associated with intelligence did not significantly alter most of the genetic correlations with a few exceptions. With intelligence controlled for, the significant genetic correlations of occupational creativity and creative achievement with autism became non-significant; the genetic correlation between creative achievement and longevity also became non-significant. Such findings suggest that the observed significant genetic correlations between creativity and autism as well as longevity may be shaped mostly by their genetic overlap with intelligence.

After partialling out genetic influences related to intelligence, the previously observed significant difference in genetic correlations across the three types of occupational creativity with other variables generally did not change significantly; The changes mainly either involved genetic correlations for scientific creativity or for well-being and success variables heavily affected by intelligence.

Our results (See Methods; Tables S16 and S17; Fig. 3) show that partialling out genetic variance associated with educational achievement, a number of the significant genetic correlations with other variables became non-significant, but not for other significant genetic correlations, for example, with risk tolerance, height, income, job satisfaction, and occupational status, for instance. In summary, the findings show that the genetic correlations of occupational creativity with the other variables cannot be entirely attributed to the genetic overlap with intelligence or educational achievement.

## Discussion

Using a large-scale GWAS approach, we investigated the genetic architecture of occupational creativity and distinguished three important forms of creativity: artistic, scientific, and managerial. We further probed the genetic correlations of occupational creativity with psychological traits, health and well-being variable, and career success. Our findings suggest the three types of occupational creativity are associated with distinct genetic variants and shared different genetic overlaps with theoretically relevant psychological traits. Furthermore, occupational creativity bore different genetic overlaps with different well-being and career success variables, suggesting some possible paradoxical influences of genetic variants associated with creativity on important career and life outcomes.

Our study identified some associated novel genes. We also found that different genes were associated with different types of creativity (e.g., artistic, scientific, and managerial), which one may use as further evidence that creativity is domain specific. Yet, we caution against such a simplistic explanation, because the different findings might also be caused by chance and the statistical power was limited so that one cannot expect the same loci to be significant in different GWASs.

In addition to corroborating prior findings of twin studies that intelligence, risk tolerance, and openness share the same genetic endowments with creativity^30,56^, our research revealed positive genetic correlations between occupational creativity and physical height and negative genetic correlations with neuroticism. Physical height and neuroticism have received little research attention in the creativity literature. Organizational research suggests that taller employees are likely to be perceived as more competent, intelligent and attractive, which in turn may bring about more advantages to come up with and experiment novel ideas^54^. Less neurotic employees are emotionally more stable, which in turn may also be beneficial for employees to implement new ideas^30^. Note that findings on significant genetic correlations do not imply causal relationships from one variable to the other. Such significant genetic correlations may also be caused by assortative mating^57^. Future research should look into these phenotypic correlations and their genetic linkages in greater depth.

Findings of the genetic correlations between occupational creativity and well-being variables suggest that there are positive genetic overlaps between creativity with both positive and negative well-being indicators. Interestingly, occupational creativity had positive genetic correlations with autism, longevity, subjective well-being, and age at first sexual intercourse. Given the mixed findings on the phenotypic relationship between creativity and autism^58^, our finding contributes to the literature by revealing that occupational creativity and autism may share the same genetic make-up, suggesting that environmental influence may play an important role in shaping their phenotypic correlation. We also found that occupational creativity had negative genetic correlations with ADHD, and BMI— indicators of low levels of well-being. Together, our findings suggest that the genetic variants positively correlated with occupational creativity may also enhance the chance for employees to have low levels (e.g., autism and bipolar disorder) and high levels of well-being (e.g., longevity and subjective well-being).

Perhaps the most intriguing results of this research lie in our revealing genetic correlations between occupational creativity with career success, which may be related to how creativity was measured in this research. Contrary to the stereotype^22,23^, we found positive genetic correlations between occupational creativity with income and occupational status. Genes positively related to occupational creativity may also enhance the chance for employees to earn high levels of income and have high status occupations—indicators of greater extrinsic career success. It is interesting that we observed a significant positive genetic correlation with only overall occupational creativity, but not with creative achievement. Considering the likely negative phenotypic correlation between creativity and income^24,25^ and the phenotypic positive relationship between creativity and job satisfaction^27^, the findings suggest that environmental influences may play a crucial role in shaping such relationships at the phenotypic level. With respect to reproductive success, occupational creativity had a positive genetic correlation with age at first birth and a negative genetic correlation with number of children. Genetic variants positively related to occupational creativity may reduce the chance for people to have high levels of reproductive success. This offers some clues to answer the age-old question: would creative genius “die out” over time^17^? Interestingly, a recent study^59^ found unique genetic variants related to creativity may partially explain why modern Homo sapiens survived and dominated over other hominids (e.g., Neanderthals and chimpanzees) in human evolution. Such seemingly paradoxical genetic influences merits future research attention.

The different genetic correlations of the different types of occupational creativity with other variables point to the importance of differentiating creativity into different domains. We found that scientific creativity shared more genetic overlap with intelligence than artistic and managerial creativity^38,39^. Managerial creativity had a higher genetic correlation with risk tolerance than artistic and scientific creativity, which is in consistent with findings of organizational research that risk tolerance is important for management and leadership roles^60^. Managerial creativity bore a higher positive genetic correlation with subjective well-being than artistic creativity. Taken together, the findings seem to suggest that compared to artistic and scientific creativity, genetic variants associated with managerial creativity appear to be related to better well-being outcomes.

We observed different genetic correlations with career success variables across the three types of occupational creativity. Artistic creativity had a lower positive genetic correlation with income than managerial and scientific creativity. Scientific creativity had a higher positive genetic correlation with age at first birth than the other two forms of creativity, but its negative genetic correlation with number of children was higher than managerial creativity. Although genetic variants related to creativity tend to reduce the chance for people to have children, this tendency seems more salient for scientific creativity.

Our GWAS results indicate that creativity phenotypes are significantly heritable traits but are highly polygenic. This complexity presents substantial challenges in accurately estimating the true “causal” effect sizes of variants within the discovery dataset, thereby reducing the efficiency of PGS using these effect estimates. Our findings underscore the polygenicity of the genetic architecture underlying creativity phenotypes.

We were unable to ascertain the causal effects of genetic variants and the direction of causality between occupational creativity with psychological traits and well-being and success variables. Psychological and organizational research on the phenotypic relationships suggests that it is possible that psychological traits and well-being variables that share the same genetic architecture with creativity may influence occupational creativity^3,16,32^. It appears also possible that creative achievement in one job may also affect job incumbents’ well-being and career success^32,61^. Our findings may also be affected by the way that occupational creativity was coded and measured. For example, one person may exhibit multiple forms of creativity (e.g., artistic and scientific simultaneously), and there are other forms of creativity beyond the three types. Therefore, future research may examine such direction of causality issues and the neurobiological pathways linking the genetic variants related to creativity measured with other approaches to brain functions, psychological traits, and well-being and success.

## Materials and Methods

### Study samples

Our main analyses were based on the U.K. Biobank (UKB) data, which were drawn from a population-based study of about 500,000 participants who were 40 years old or older in the United Kingdom^42,62,63^. Participants’ information on their jobs and occupations was obtained through interviews during their visits to the study centers. UKB staffs coded such job and occupation information (e.g., job titles and major responsibilities) into the four-digit U.K. Standard Occupational Classification (SOC) system^37^ (version 2000; Field identifier: 132). Our analyses included participants of European ancestry with available information on their occupation codes and genotype data that passed quality control (Figure S7).

Replication samples included participants from the UKB follow-up cohort (*N* = 23,249), the Add Health Wave IV cohort^64,65^ (*N* = 1461), and Wisconsin Longitudinal Study (WLS) cohort^66^ (*N* = 2265). Between June to September 2015, about 102,000 UKB participants completed an online follow-up assessment of their employment history. We selected those who had past employment information but were not included in the discovery phase because of lacking baseline job information. The Add Health study is a U.S. longitudinal study of adolescents^67^. We used data from the Add Health Wave IV survey that was conducted between 2007 to 2009, and included unrelated Caucasian participants with valid occupation information and genotype data in analyses. The Wisconsin Longitudinal Study (WLS) cohort is a large U.S. longitudinal study of a random sample of people who graduated from Wisconsin high schools in 1957 and of their randomly selected siblings^66^. Six rounds of data collection were conducted from 1957 to 2011. The occupation information was collected in a random sample in the last four rounds. More detailed information about the data preparation of the replication samples is presented in Supplementary notes and Figures S8-10.

### Phenotype definitions and measures

Because multifaceted nature, creativity has been very challenging to measure and thus it has been assessed in various fashions in the literature^13,30^. In this research, we adopted the previous approach^8,38,68^ and captured creativity primarily based on job tasks and responsibilities of participants’ occupations with two measures (five variables, Table S1). The first measure was categorical, encompassing an overall measure of occupational creativity and its three domain-specific types: artistic creativity, scientific creativity, and managerial creativity. The second measure was a continuous variable, creative achievement, which served as a common metric of creativity across various occupations.

#### Occupational creativity

The first measure of occupational creativity was derived from the major responsibilities of participants’ occupations either from the U.K. SOC 2000 occupational codes (for the UKB data) or from the U.S. Occupational Information Network (O*NET, https://www.onetonline.org/) occupational codes (for the WLS and Add Health data). Specifically, based on the literature on creativity^13,14^, we identified three groups of occupations from the U.K. SOC 2000 or the U.S. O*NET systems that require high levels of creativity (i.e., artistic, scientific, and managerial) and one control group (i.e., conventional occupations) that require low levels of creativity based on the major tasks and responsibilities of participants’ occupations. This generated four binary variables with three reflecting the three domain-specific creativity (e.g., for artistic creativity, 1 = artist occupation and 0 = conventional occupation). For the fourth variable reflecting overall occupational creativity, we coded all the artistic, scientific, and managerial occupations as creative occupations (i.e., as 1) and used less creative/conventional occupations as the reference group (coded as 0). Below we provide a detailed description of how each group of domain-specific creativity was operationalized. For the control group, we drew from Holland’s research on occupational interest^69^ and coded conventional occupations as those with low levels of creativity, because their primary tasks are more structured, orderly, and routinized, and thus require less novel ideas^69,70^. Sample occupational titles of the control group include counter clerks, plastics process operatives, and filing assistants/clerks. Occupations that do not clearly fall into the four categories of artisitic, scientific, manegrial, and conventional jobs were coded as missing values.

#### Artistic creativity

Artists have long been treated as creative professionals^14^. Following previous research on artistic occupations^8,71^, we included the following occupations as those with high levels of artistic creativity: actors, dancers, entertainers, musicians, visual artists, choreographers and writers.

#### Scientific creativity

Prior research has also identified scientific occupations as creative occupations^8,71^, because scientists are required to generate innovative and useful ideas in conducting academic research. Such scientific occupations are not limited to those in natural and biological sciences, but also include those in social sciences, engineering, and mathematics. We thus operationalized scientific creativity as those occupations whose primary job tasks and responsibilities involve undertaking research in various sciences and conceiving engineering designs. Sample occupation titles were chemists, biological scientists, civil engineers, and scientific researchers.

#### Managerial creativity

Drawing on previous literature on creativity and leadership^28,29^, we proposed a new category of creative professions: managerial occupations. We treated senior managers and officials as creative occupations because incumbents of such occupations are increasingly required to generate novel ideas, practices, and solutions to direct and coordinate the functioning of organizations and work teams, particularly when faced with challenges and uncertainties brought about by the changing business environment^28^. Sample occupation titles include directors and chief executives of major organizations, marketing and sales managers, and senior officers in fire, ambulance, prison and related services.

To ensure the reliability and validity of our coding, we developed a coding scheme based on previous literature that defined four occupation groups^8,69,71^. Two independent raters coded all the occupations by carefully reviewing the structure of U.K. SOC 2000, the U.S. O*NET and SOC systems, and the detailed descriptions of the tasks and duties of each occupation described in the occupation classification systems, as well as the cross-country crosswalk on the basis of International Labor Organization’s International Standard Classification of Occupations (ISCO-88 and ISCO-08) to ensure accuracy and reliability of the coding results. Furthermore, the raters also referred to the list of creative industries and occupations released by the U.K. Department for Digital, Culture, Media and Sport^72,73^ to ensure that their coding was consistent with the major consensus in the creative industry in the world. The initial interrater agreement was approximately 90%. Discrepancies were resolved through discussion among all the raters and authors.

#### Creative achievement

Our second measure of creativity—creative achievement—was assessed with three items obtained from the U.S. O*NET database with a crosswalk by linking the U.K. SOC 2000 occupation codes in the UKB data to the occupation codes in the O*NET system. For the Add Health and WLS data, given that participants’ occupation codes were based on the U.S. SOC system, we extracted information on creative achievement from O*NET directly by matching participants’ occupation codes with the O*NET occupation codes. The three items tap into core characteristics of creativity: fluency of ideas, originality, and thinking creatively. Sample items include “What level of thinking creatively is needed to perform your current job?” and “What level of originality is needed to perform your current job?” All the items used a seven-point scale, ranging from the lowest (1) to the highest (7) level of creative achievement. The internal consistency of this scale (Cronbach’s α) was 0.92.

### Genotyping and imputation

We used imputed genotypes released by U.K. Biobank (bgen files; imputed data v3 – released in March 2018). The quality control and imputation were done by U.K. Biobank^42^. Briefly, genotyped variants were filtered based on batch effects, plate effects, departures from Hardy Weinberg equilibrium (HWE), genotype platform, and discordance across control replicates. Participant samples were excluded based on missing rate larger than 5%, inconsistencies in reported versus genetic sex, and excessive heterozygosity based on a set of 605,876 high quality autosomal markers. Genotypes were phased and the imputation was performed using IMPUTE4 with the Haplotype Reference Consortium (HRC) data, UK10K and 1000 Genomes Phase 3 dataset used as the reference set. A European subset was identified by projecting the UKB participants onto the 1000 Genome Project principal components coordination. For this study, we excluded genetic variants with MAF < 1%, and poorly imputed markers (IMPUTE info < 0.3), resulting in 9,804,641 autosomal variants imputed or genotyped on individuals of European ancestry. The genotyping, imputation, and filtering procedure were similar across the UKB discovery sample and the follow-up sample.

For the Add Health cohort, the genotypes were imputed on the Haplotype Reference Consortium, with quality controls detailed previously^74^. Genetic variants were included with MAF > 1% and IMPUTE Info < 0.3. Analyses were limited to individuals of European-ancestry and cryptically related individuals were dropped from analyses. For the WLS cohort, the genotypes were imputed using the 1000 Genomes Phase 3 dataset. Replication analyses included only unrelated European participants.

### Genome-wide association analyses

We assumed an additive genetic model where the dosage of each SNP was a continuous variable ranging from 0 to 2 for the effect allele. For UKB GWAS, a linear mixed model accounting for genetic relatedness was conducted to determine its association with the phenotypes. The analyses were conducted with the software BOLT-LMM v.2.3.2 (https://data.broadinstitute.org/alkesgroup/BOLT-LMM/downloads)^75^. The association analyses were adjusted for age, sex, genotyping array, and top 20 principal components (PCs). The GWASs were also conducted separately by sex.

Significant independent variants and their surrounding genomic loci were identified using LD-clumping in PLINK v.2.00 (https://www.cog-genomics.org/plink/2.0/). The LD structure was estimated from the European panels in the 1000 Genome Project of phase 3 as the reference population. For GWAS conducted for 5 traits in this study, we set *P* < 1 × 10^−8^ as the genome-wide significance. The index lead SNP was identified (*P* < 1 × 10^−8^), independent from other variants (*r*^*2*^ < 0.01), at each locus. Here a locus was defined by an index SNP with the region flanking 500 kb on both sides. The coordinates and variant identifiers were reported on the NCBI B37 (hg19) genome build. The functional annotation and gene mapping were performed using ANNOVAR (v.2018Apr16, https://doc-openbio.readthedocs.io/projects/annovar/en/latest/user-guide/download/), including types of intronic, exonic, intergenic, 5’-UTR, and 3’-UTR, etc.^76^. Regional plots of each identified locus were made by LocusZoom (http://locuszoom.org/), and the 1000 Genome of the European population was used to estimate LD information.

### Replication analyses

Genome-wide significant SNPs at the top loci were evaluated in the UKB follow-up dataset, the Add Health dataset, and the WLS cohort. We meta-analyzed results with both discovery and replication samples using the inverse-variance weighted fix-effect model with METAL software (https://genome.sph.umich.edu/wiki/METAL). For the four binary creative variables (artistic, scientific, and managerial, and overall creativity), the coefficients obtained from the linear mixed model in the UKB discovery sample were on a standardized scale. Therefore, we transformed these coefficients to make them comparable with the observations in all samples. We rescaled the beta coefficients with the following formula: *β*_*s*_ = *β* / k*(1 − *k*), where *k* is the prevalence of creative occupations in the UKB; the ORs were calculated accordingly using the scaled beta coefficient *β*_*s*_.

### Common SNP heritability

We employed the software LD-score regression (LDSC) v.1.0.1 (https://github.com/bulik/ldsc) with GWAS summary statistics in our estimation of common SNP*-h*^*2*^ for the creativity phenotypes for the whole sample and sub-samples by sex in the UKB discovery data^52^. We used LDSC to estimate the proportion of variance in liability to creativity traits that was explained by the aggregated effect of the SNPs. From GWAS summary-level data, we included SNPs that were presented in the European panels in the 1000 Genome Project, with the exclusion of the major histocompatibility complex (MHC) region on chromosome 6. SNPs with INFO < 0.8 were not included in the LDSC regression analyses.

For the binary trait of creative occupations (artistic, scientific, and managerial, and overall creativity), the estimated heritability was transformed to the liability scale using the approach derived by Lee et al.^77^. As the exact prevalence is unknown, we assumed the percentage of creative occupations in the UKB sample under current analysis was equal to the population prevalence, an approach adopted in calculating SNP*-h*^*2*^ on the liability scale for other dichotomous traits using the UKB data (http://www.nealelab.is/uk-biobank**)**.

### Genetic correlations of occupational creativity with personal, well-being, and success variables

We computed the genetic correlation among creativity variables (for the overall sample and sub-samples by sex) using the GWAS summary statistics from the UKB discovery sample. We adopted the bivariate LDSC method, by regressing the product of testing statistics (z statistics) from each phenotype against the LD scores^78^.

We assessed the genetic correlations of creativity phenotypes with personal traits, well-being, and success variables using summary statistics from GWASs of European ancestry, with the detailed information of sample sizes, phenotypes, and GWAS summary data resources presented in Table S11. We used summary statistics from the previously published large-scale GWAS or GWAS results based on the UKB data, including, for example, subjective well-being^79^, job satisfaction (UKB data 4537), depressive symptom^79^, neuroticism^80^, longevity^81^, number of cancer illness (UKB data 134), number of noncancer illness (UKB data 135), BMI^82^, height^82^, and intelligence^83^. Note that for the illustration purpose, we recoded job satisfaction so that higher scores indicate higher levels of job satisfaction.

To estimate the genetic correlations after partialling out the genetic variance of intelligence or educational attainment, we used the Genomic SEM^55^. For each genetic correlation between creativity and other phenotypes, we fitted the Genomic SEM model including three traits: creativity trait (X), the other phenotype (Y), and intelligence (Z). In the path diagram, there was a bidirectional arrow between two traits of X and Y, and uni-directional arrows from Z to X and Z to Y. The genetic effect of Z was regressed out from the variance of X and Y affecting the genetic correlation. The genetic covariance matrix of X, Y, and Z was produced by the LDSC method implemented in Genomic SEM. The process was repeated for each of the personal traits, well-being and success variables.

### Polygenic scores (PGS) analyses

Using the GWAS results from creativity traits, we generated PGSs in the three replication samples. The polygenic scores were constructed in PRSice (see URL: https://choishingwan.github.io/PRSice), a method shown to have decent prediction accuracy involving LD pruning followed by p-value thresholding^50^, and PRS-CS (see URL: https://github.com/getian107/PRScs), a method which generates posterior SNP effect size estimates using Bayesian regressions with continuous shrinkage priors^51^. For the PRSice method, besides automatically optimizing p-value cutoff as default, variants were also selected at p-value thresholds: *p* < 0.01 and *p* < 1 × 10^−4^. Independent lead variants associated with the phenotype were identified by the “clumping and thresholding” approach, removing those within 500 kb and in linkage disequilibrium r^2^ ≥ 0.01 with the lead variant in the region. An individual’s polygenic score is a weighted sum of the genotypes across all independent variants. The weighting factor is the estimated additive effect size, beta coefficient, at each variant from the GWAS summary statistics. Prediction accuracy was based on an ordinary least squares (OLS) regression of the creativity phenotypes on the polygenic score and a set of standard covariates, including age, sex, and the top genetic PCs. The R^2^ or McFadden pseudo-R^2^ (for binary outcome) for PGS was calculated as the incremental variance for creativity variables, i.e., the R^2^ of the model including polygenic scores and covariates minus the R^2^ of the model including only covariates. For the PRS-CS method, the 1000 Genomes Project Phase 3 European sample is used as the external LD reference panel. The parameters in the gamma-gamma prior are set as 1 and 0.5 respectively and the global shrinkage parameter is automatically learned from GWAS summary statistics using a fully Bayesian approach. The posterior SNP effect size estimates were concatenated across all chromosomes, which was used as weight for calculating individual’s polygenic score in each cohort.

### Reporting summary

Further information on research design is available in the Nature Portfolio Reporting Summary linked to this article.

## Supplementary information

Supplementary Material

Reporting summary

## Data Availability

The UKB GWAS summary statistics for the five creativity phenotypes will be available on the NHGRI-EPI Catalog of human GWAS upon publication. The accession codes for occupational creativity, artistic creativity, scientific creativity, managerial creativity, and creative achievement are GCST90444391, GCST90444392, GCST90444393, GCST90444394, GCST90444395, respectively. The summary statistics of the top index variants were presented in the article or supporting information, along with the data analyzed in this study.
