# Supplementary material for: A genome-wide association study of occupational creativity and its relations with well-being and career success
Source: Commun Biol. 2024 Sep 5;7:1092. doi: 10.1038/s42003-024-06686-5 (PMC11377709; doi:10.1038/s42003-024-06686-5)
Supplement: Supplementary file 1 — Supplementary Material [file 42003_2024_6686_MOESM1_ESM.pdf]

A Genome-Wide Association Study of Occupational Creativity and its Relations with Well-Being and Career Success

Supplementary Materials

Supplementary Figures

Figure S1. Distribution of creativity phenotype by sex

Figure S2. Manhattan plot of GWAS analysis for three types of creativity in the UKB discovery sample (a) artistic creativity; (b) scientific creativity; (c) managerial creativity

Figure S3. Quantile-quantile plots with genomic control inflation factor lambda λ_G (a) artistic creativity; (b) scientific creativity; (c) managerial creativity; (d) overall occupational creativity; (e) creative achievement

Figure S4. Quantile-quantile plot of GWAS meta-analysis results for creativity across UKB, WLS, and the Add Health samples (a) artistic creativity; (b) scientific creativity; (c) managerial creativity; (d) overall occupational creativity; (e) creative achievement

Figure S5. Manhattan plot of GWAS analysis (a) artistic creativity (male); (b) artistic creativity (female); (c) scientific creativity (male); (d) scientific creativity (female); (e) managerial creativity (male); (f) managerial creativity (female); (g) overall occupational creativity (male); (h) overall occupational creativity (female); (i) creative achievement (male); (j) creative achievement (female).

Figure S6. Genetic correlations of artistic, scientific, and managerial creativity with outcomes

Figure S7. Data preparation for the U.K. Biobank discovery sample

Figure S8. Data preparation for the U.K. Biobank follow-up sample.

Figure S9. Data preparation for the Add Health Wave V data.

Figure S10. Data preparation for the Wisconsin Longitudinal Study data.

Supplementary Tables

Table S1. Phenotypic descriptions of creativity

Table S2. Summary of the creativity phenotypes in the UKB discovery sample by sex

Table S3. Summary of O*NET linked phenotype scores of occupational creativity in the U.K. Biobank discovery data

Table S4. Phenotypic correlations among creativity phenotypes

Table S5. Sample size and demographic distribution in replication samples

Table S6. Genomic control λ for GWAS on creativity phenotypes in UK Biobank data

Table S7-1. Summary of top loci for creativity traits identified from UKB data

Table S7-2. A meta-analysis of top loci for creativity variables across UKB, Add Health, and WLS datasets

Table S8. A meta-analysis of top loci for creativity variables across UKB, Add Health, and WLS datasets by sex

Table S9. Summary of common SNP heritability estimations for creativity phenotypes from GWAS results in UKB data

Table S10. Genetic correlations for creativity phenotypes across sexes in the UKB sample

Table S11. Sources and phenotype descriptions for GWAS results used in the genetic correlation analysis

Table S12. Genetic correlations between creativity and outcome variables

Table S13. Comparison of genetic correlations between creativity phenotypes and outcome variables

Table S14. Genetic correlations between creativity and outcome variables, partialling out intelligence

Table S15. Comparison of genetic correlations between creativity phenotypes and outcome variables, partialling out intelligence

Table S16. Genetic correlations between creativity and outcome variables, partialling out educational achievement

Table S17. Comparison of genetic correlations between creativity phenotypes and outcome variables, partialling out educational achievement

Table S18. Constructing PGS for creativity in UKB follow-up cohort, Add Health cohort, and WLS cohort

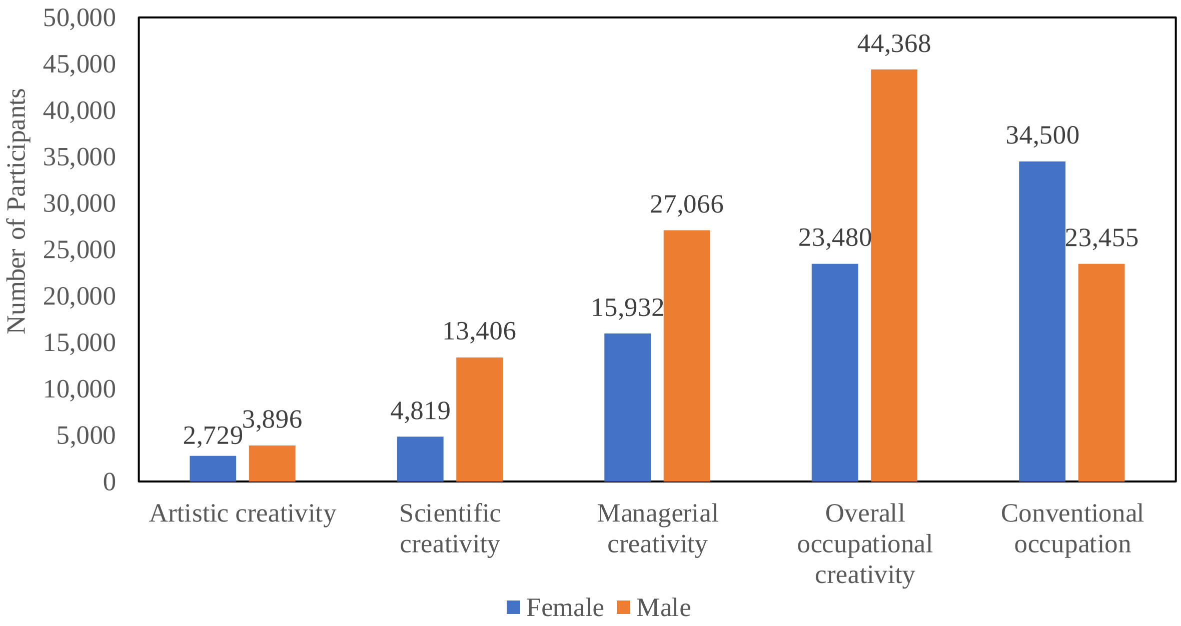

Figure S1. Distribution of creativity phenotype by sex.

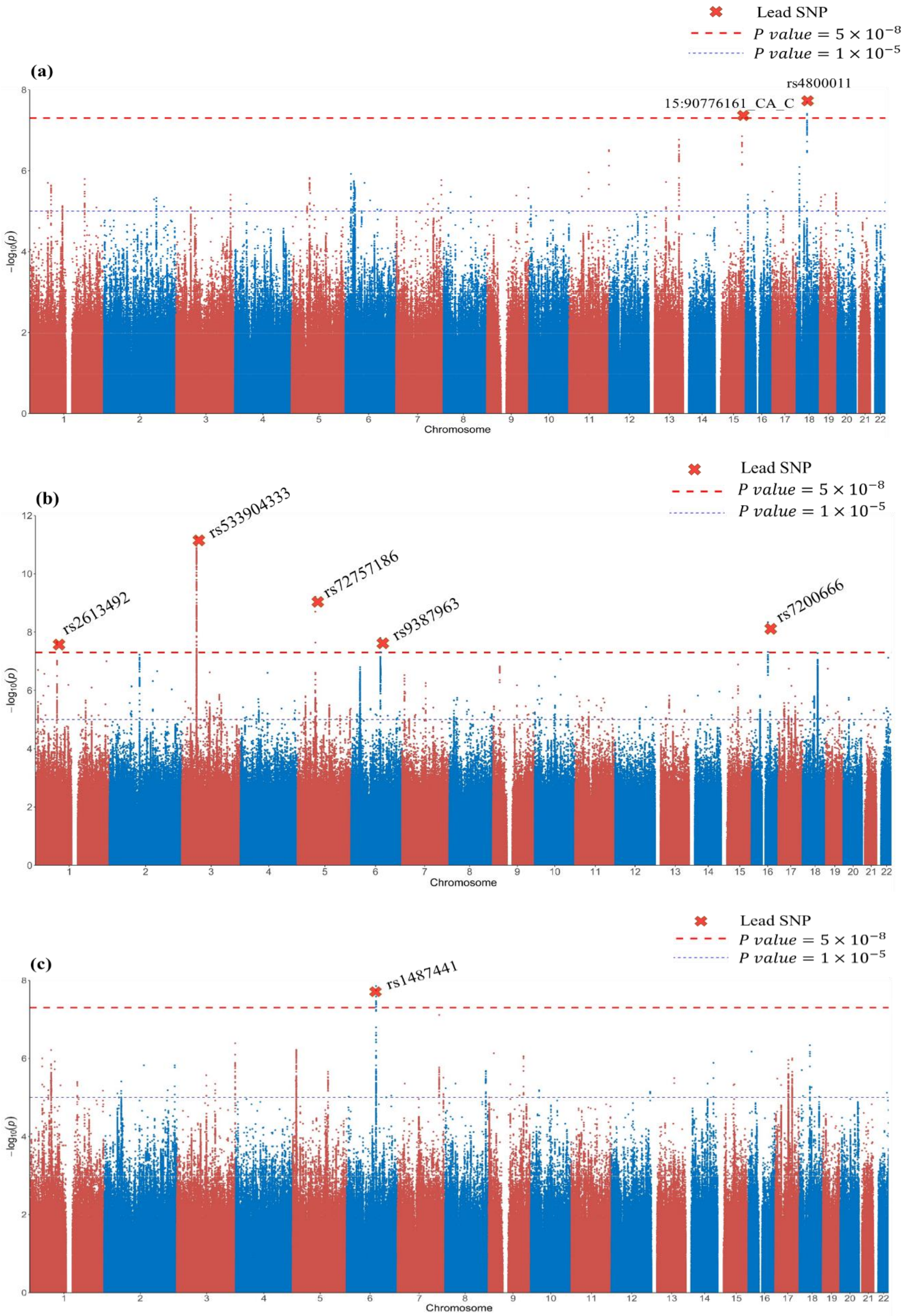

Figure S2. Manhattan plot of GWAS analysis for three types of creativity in the UKB discovery sample (a) artistic creativity; (b) scientific creativity; (c) managerial creativity.

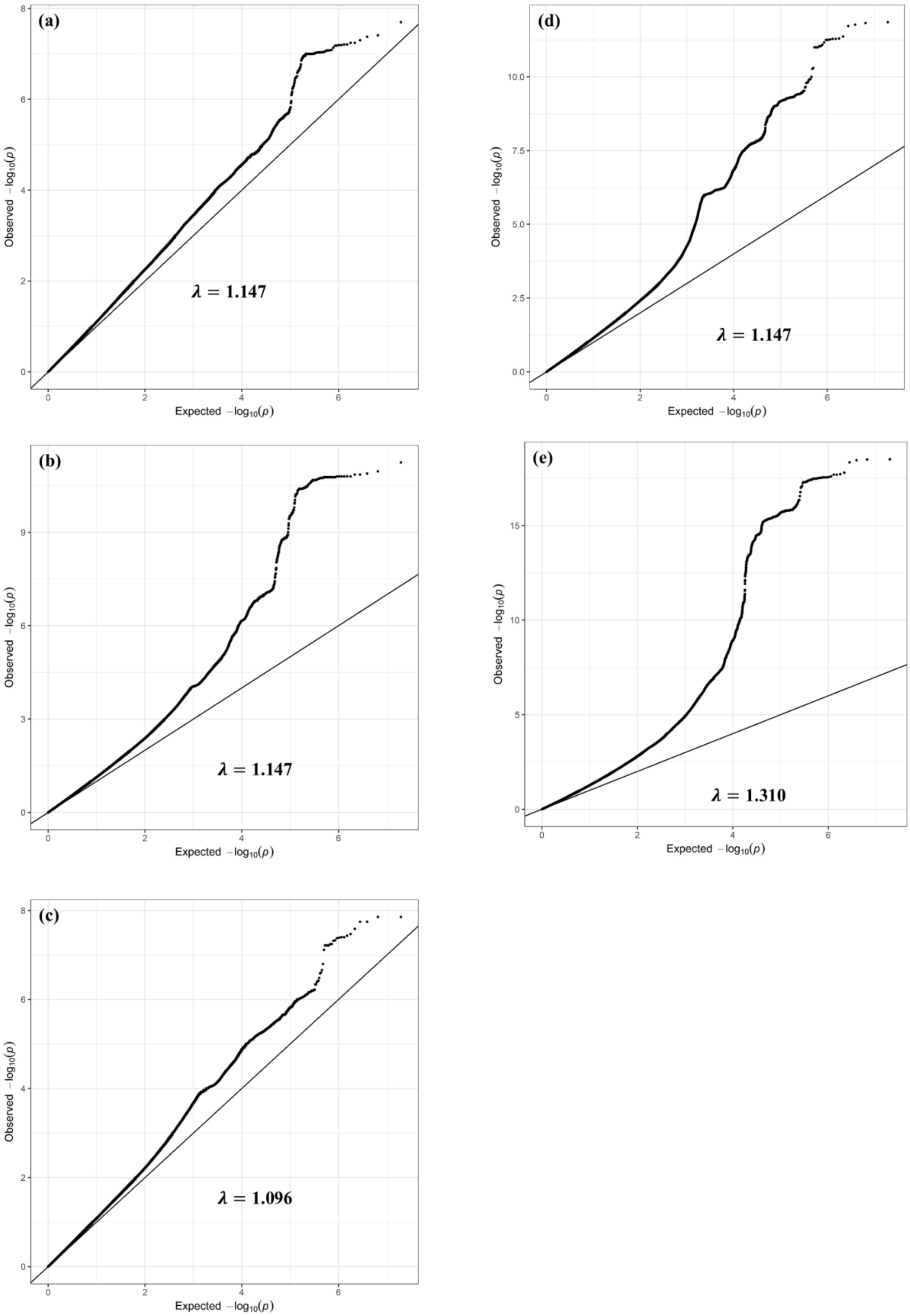

Figure S3. Quantile-quantile plots with genomic control inflation factor lambda λ_G (a) artistic creativity; (b) scientific creativity; (c) managerial creativity; (d) overall occupational creativity; (e) creative achievement.

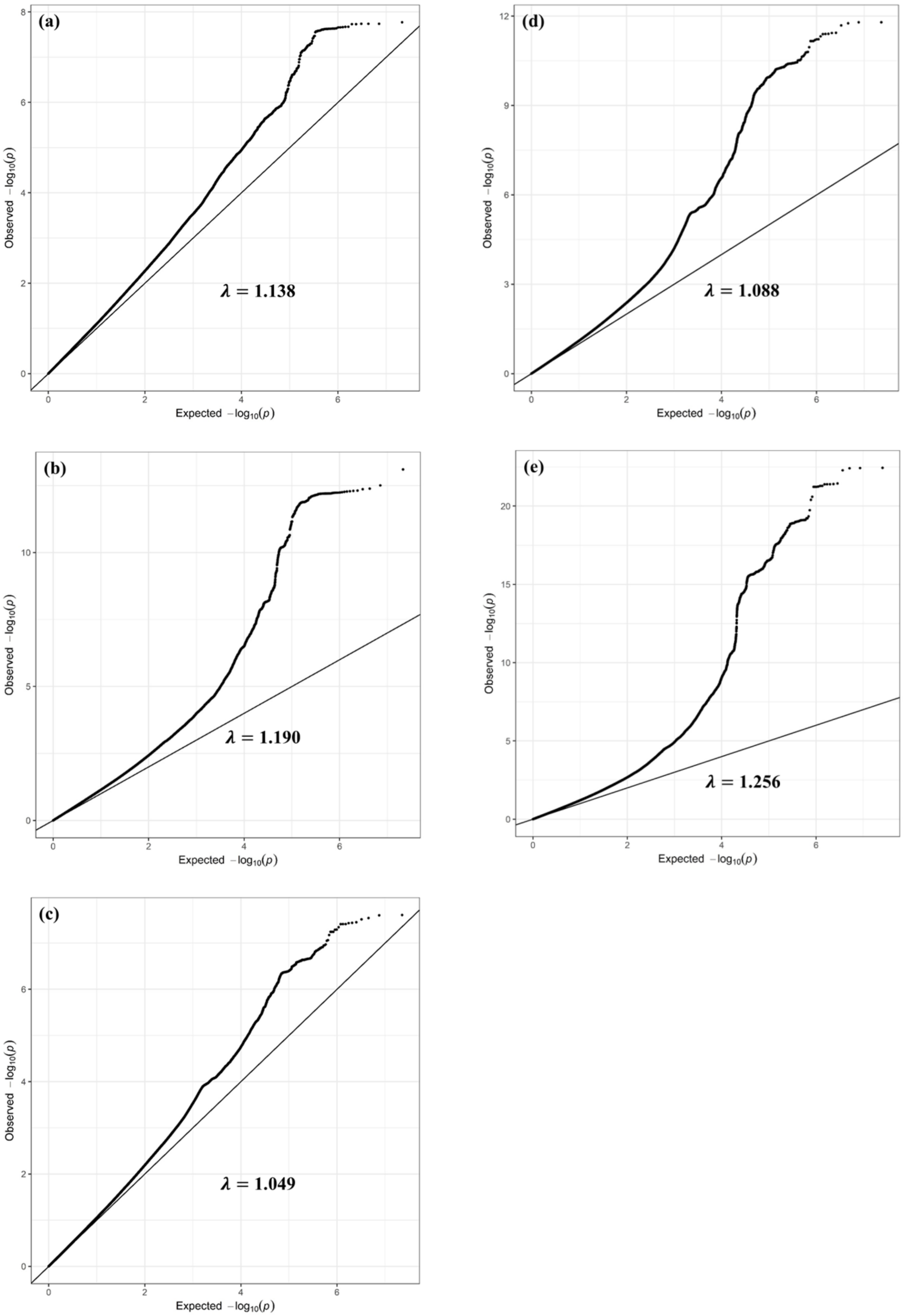

Figure S4. Quantile-quantile plot of GWAS meta-analysis results for creativity across UKB, WLS, and the Add Health samples (a) artistic creativity; (b) scientific creativity; (c) managerial creativity; (d) overall occupational creativity; (e) creative achievement.

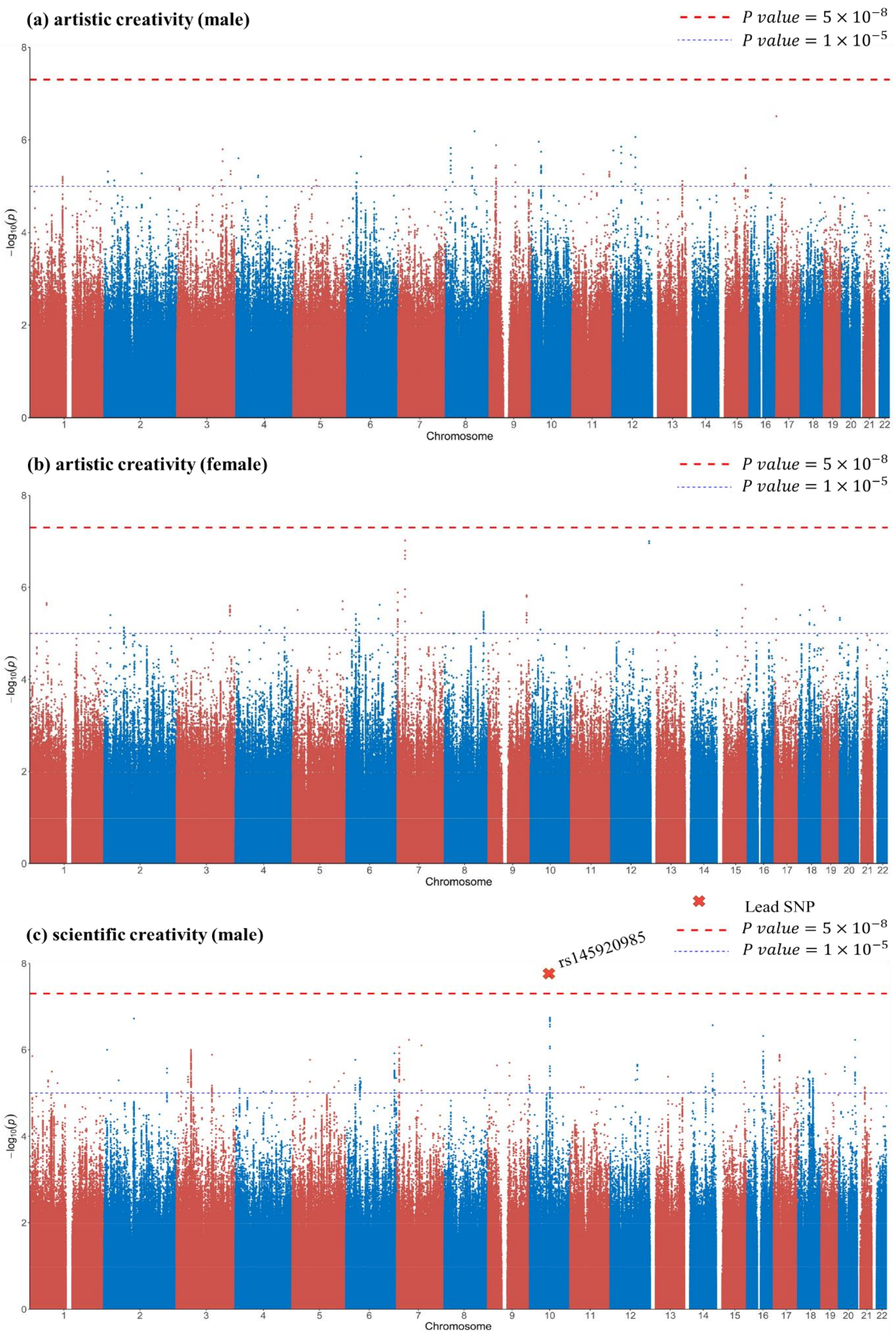
(a) artistic creativity (male)
$P\ value = 5 \times 10^{-8}$
$P\ value = 1 \times 10^{-5}$
$-\log_{10}(p)$
Chromosome
(b) artistic creativity (female)
$P\ value = 5 \times 10^{-8}$
$P\ value = 1 \times 10^{-5}$
$-\log_{10}(p)$
Chromosome
(c) scientific creativity (male)
Lead SNP
$P\ value = 5 \times 10^{-8}$
$P\ value = 1 \times 10^{-5}$
rs145920985
$-\log_{10}(p)$
Chromosome

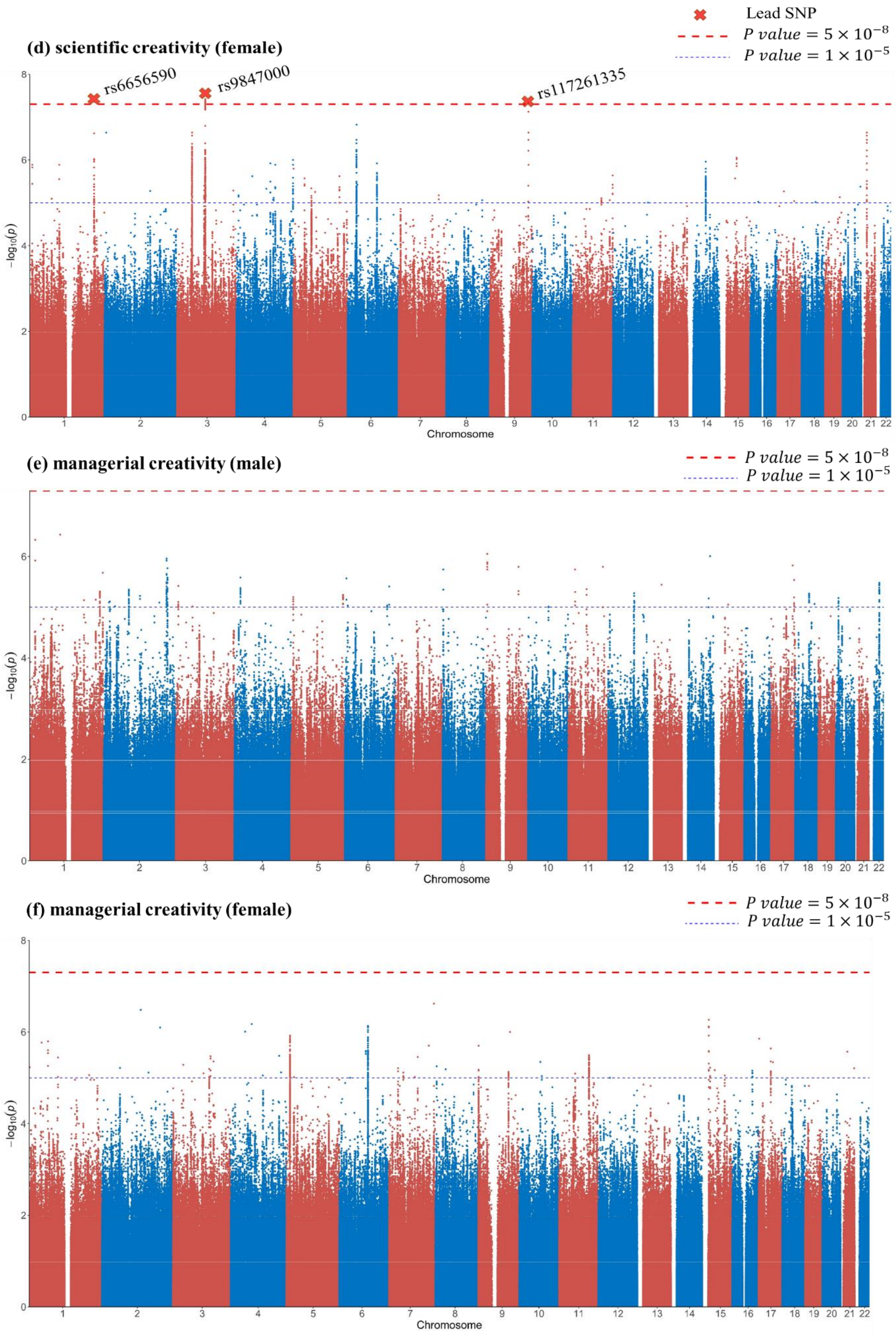

Lead SNP
$P\ value = 5 \times 10^{-8}$
$P\ value = 1 \times 10^{-5}$
(d) scientific creativity (female)
rs6656590
rs9847000
rs117261335
$-\log_{10}(p)$
Chromosome
(e) managerial creativity (male)
$P\ value = 5 \times 10^{-8}$
$P\ value = 1 \times 10^{-5}$
$-\log_{10}(p)$
Chromosome
(f) managerial creativity (female)
$P\ value = 5 \times 10^{-8}$
$P\ value = 1 \times 10^{-5}$
$-\log_{10}(p)$
Chromosome

(g) overall occupational creativity (male)

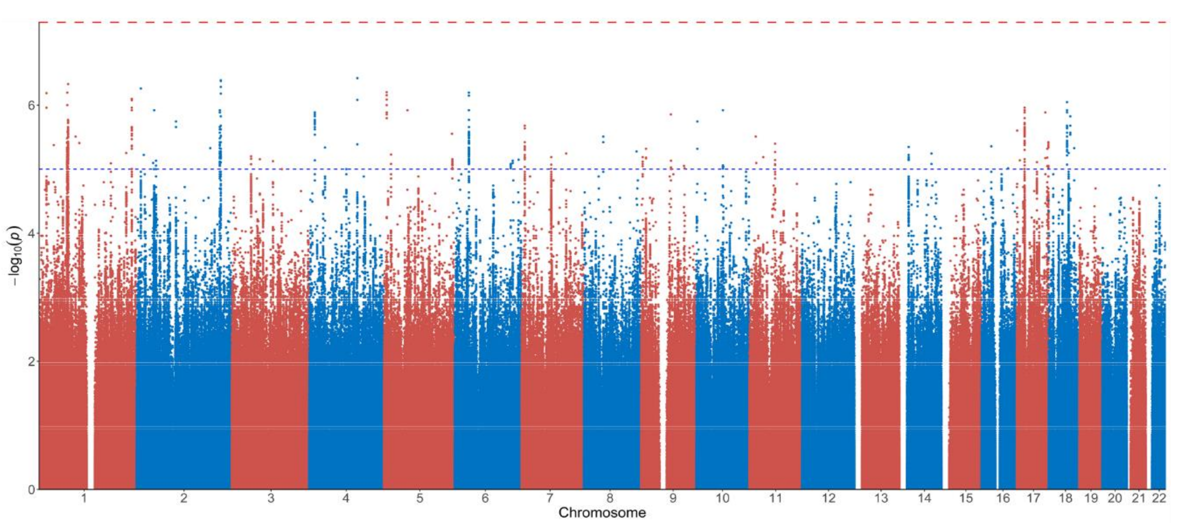

(h) overall occupational creativity (female)

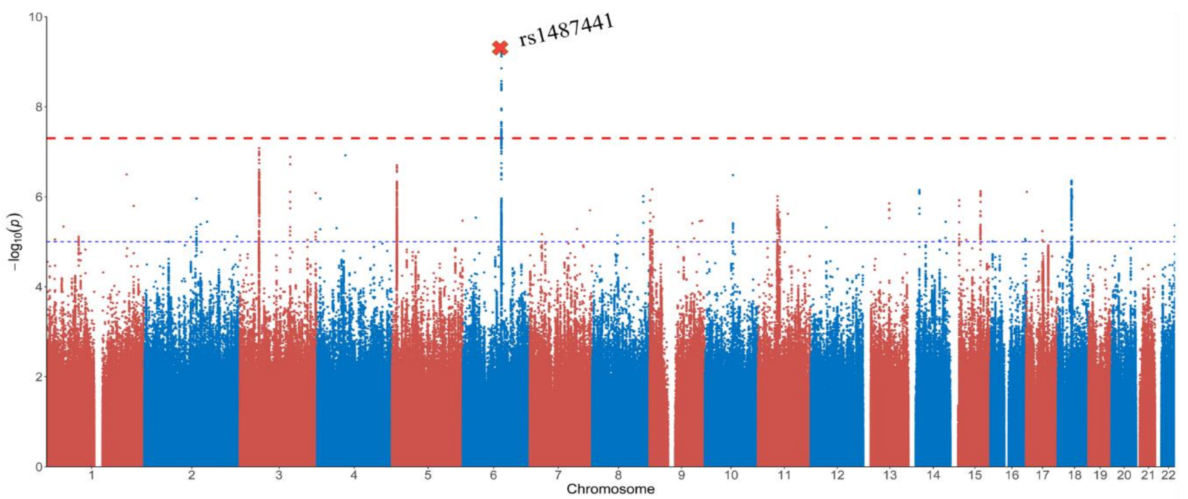

(i) creative achievement (male)

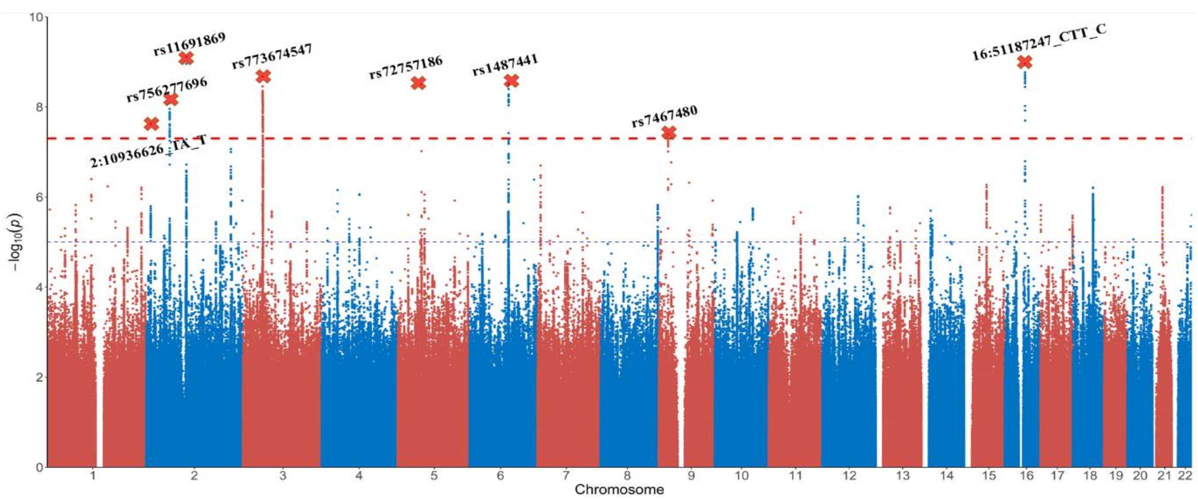

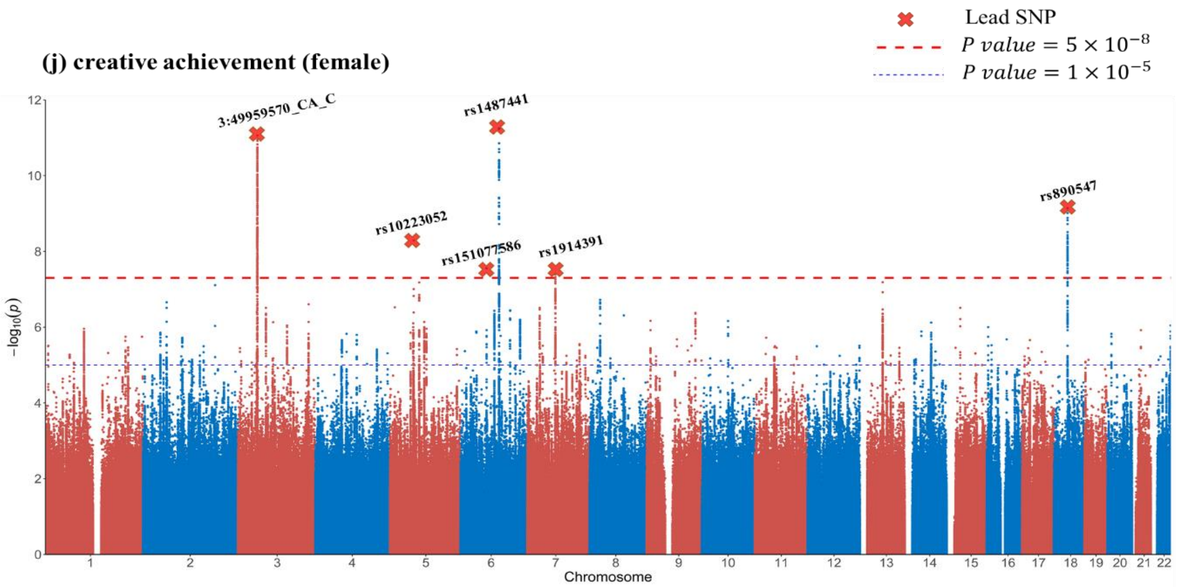

Figure S5. Manhattan plot of GWAS analysis (a) artistic creativity (male); (b) artistic creativity (female); (c) scientific creativity (male); (d) scientific creativity (female); (e) managerial creativity (male); (f) managerial creativity (female); (g) overall occupational creativity (male); (h) overall occupational creativity (female); (i) creative achievement (male); (j) creative achievement (female).

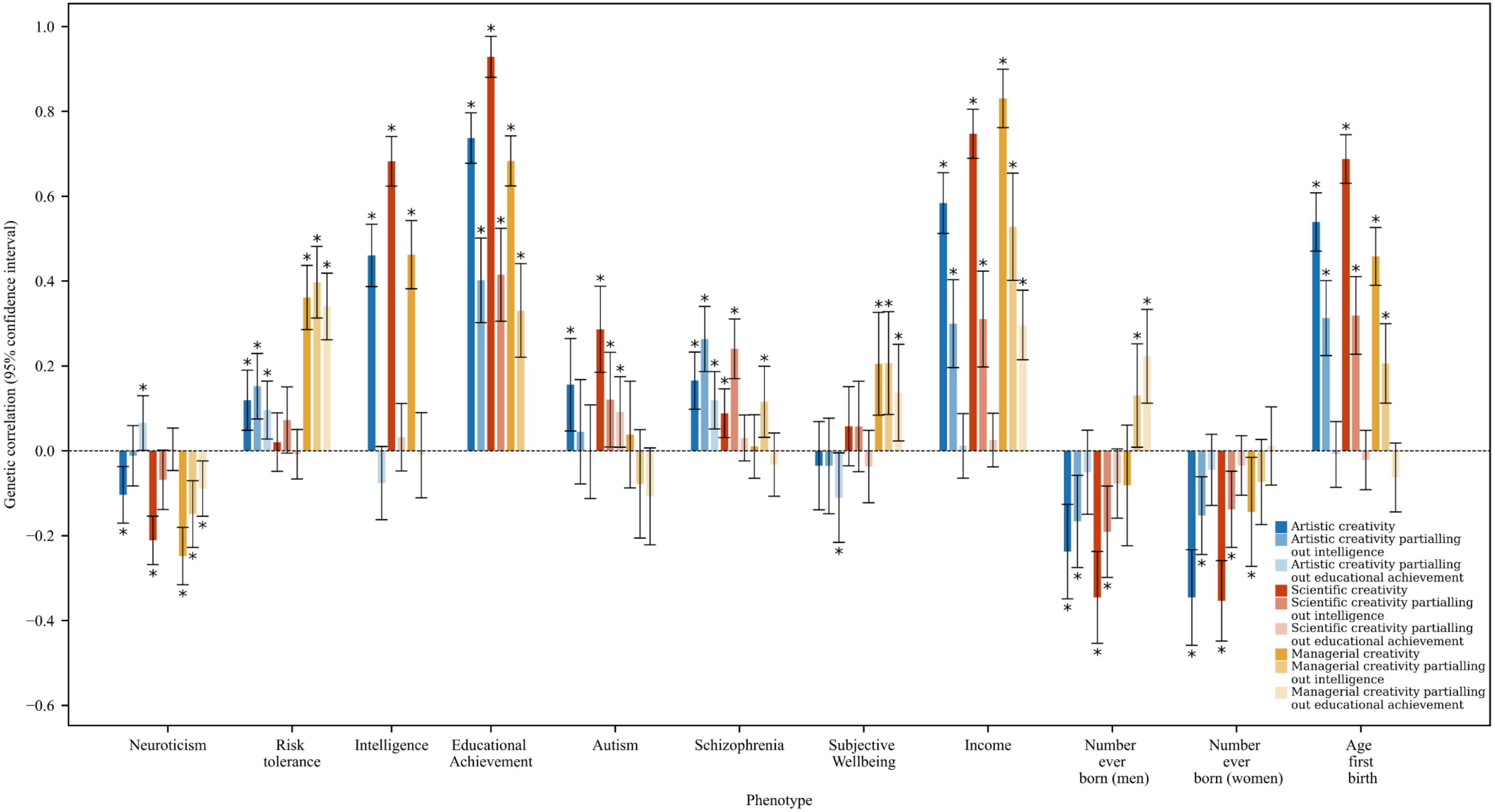

Figure S6. Genetic correlations of artistic, scientific, and managerial creativity with outcomes. Vertical bars represent 95% CIs. Asterisks denote the genetic correlations at FDR < 0.05.

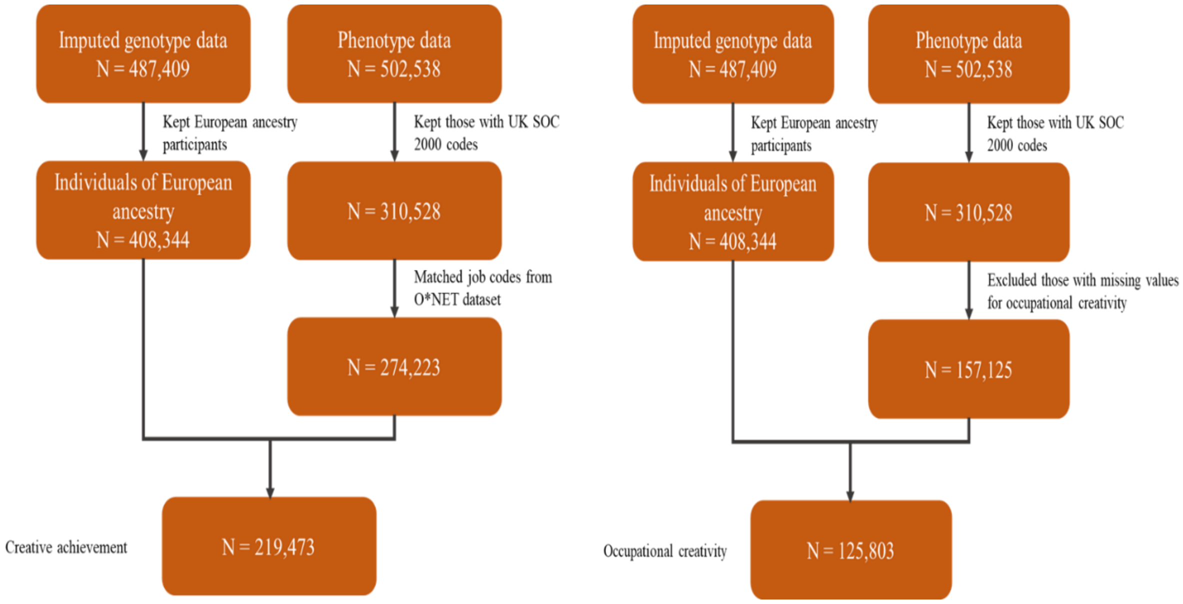

Figure S7. Data preparation for the U.K. Biobank discovery sample. The current analysis includes U.K. biobank imputed genotype data for the European population only. A total of 125,803 individuals with occupational creativitiy phenotype from UK SOC 2000 codes were included. By linking to the O*NET dataset, 219,473 individuals with creative achievement phenotypes were included in GWAS analysis.

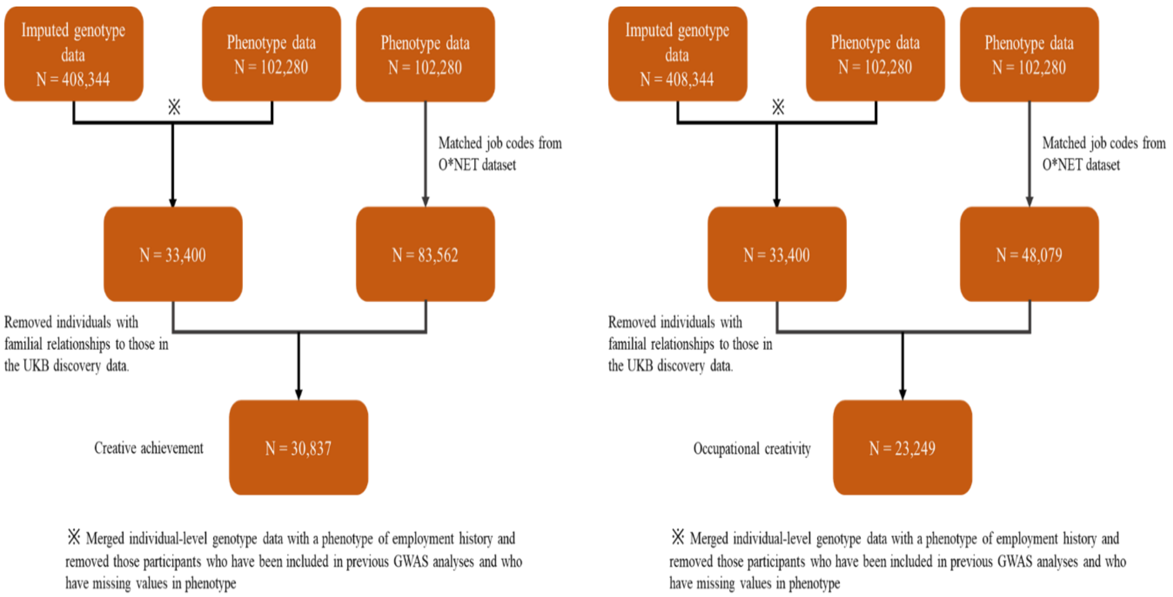

Figure S8. Data preparation for the U.K. Biobank follow-up sample. The replication samples comprised 23,249 individuals for occupational creativity and 30,837 individuals for creative achievement with genome data and valid job information of the last job, but not included in the discovery phase.

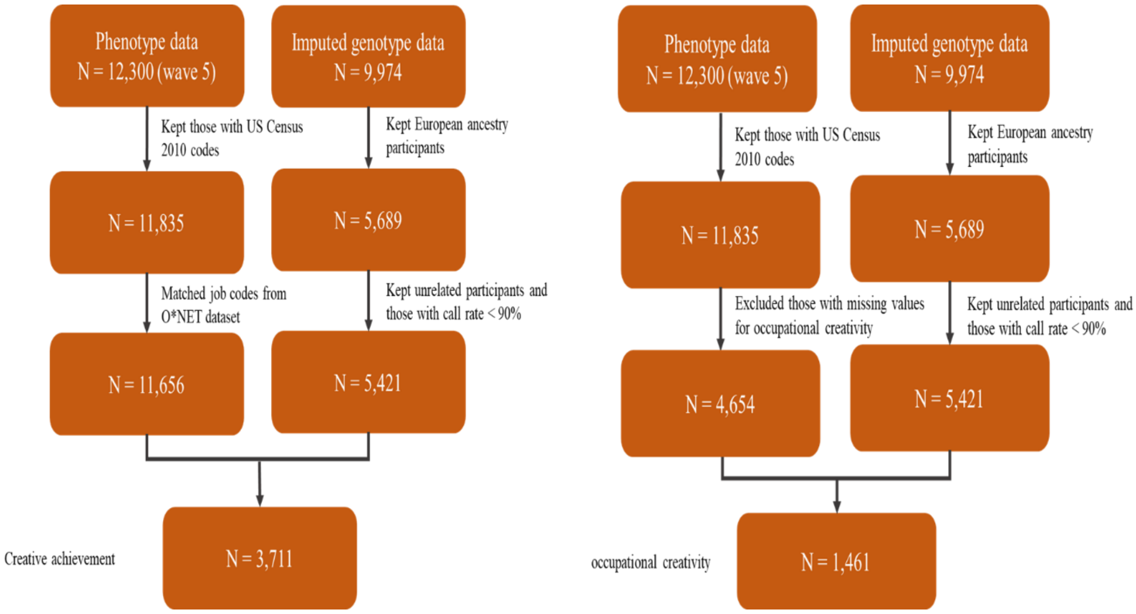

Figure S9. Data preparation for the Add Health Wave V data. The replication stage includes unrelated individuals of European ancestry from the Add Health Wave 5 data, with imputed markers, referenced to the Haplotype Reference Consortium panel (HRCr1.1). 3,711 individuals were available for creative achievement and 1,461 individuals were available for occupational creativity.

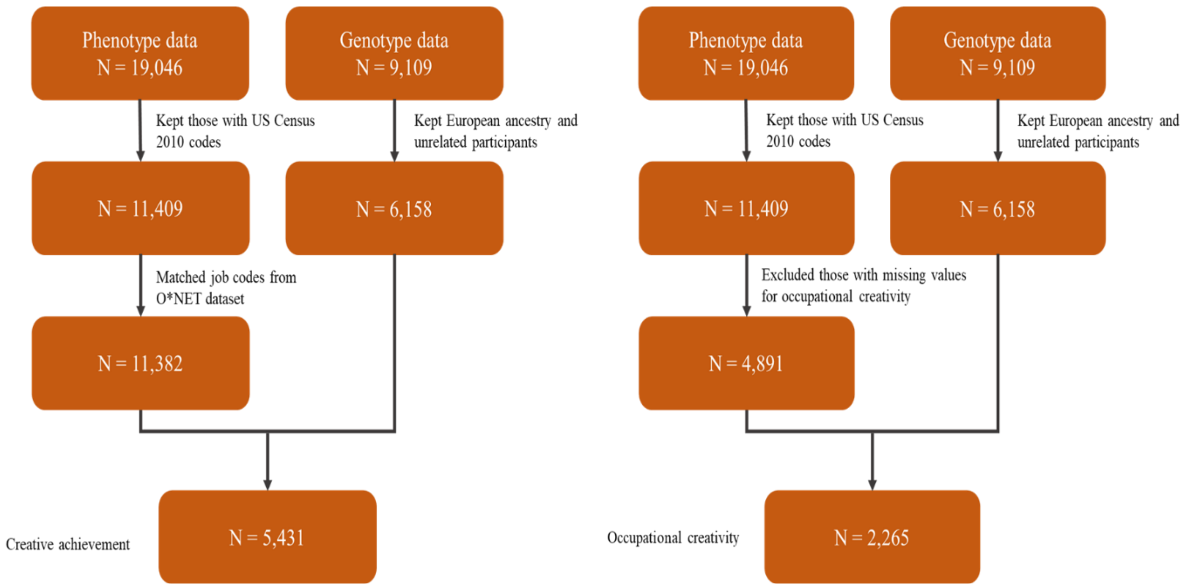

Figure S10. Data preparation for the Wisconsin Longitudinal Study data. The replication stage included unrelated individuals of European ancestry from the Wisconsin Longitudinal Study data. 5,431 individuals were available for creative achievement and 2,265 individuals were available for occupational creativity.

Table S1. Phenotypic descriptions of creativity

Variable name	Source	Description	Coding
Artistic creativity	UK SOC 2000 Job codes	We included actors, dancers, musicians, visual artists, and writers as artistic occupations (coded as 1). Sample job titles include authors and writers, actors and entertainers, and dancers and choreographers. Likewise, less creative occupations (i.e., conventional occupations) were coded as 0.	1, 0
Scientific creativity	UK SOC 2000 Job codes	Scientific occupations whose primary job tasks involve conducting research and conceiving engineering designs were coded as 1. Sample job titles are chemists, biological scientists, civil engineers, and scientific researchers. Likewise, less creative occupations (i.e., conventional occupations) were coded as 0.	1, 0
Managerial creativity	UK SOC 2000 Job codes	Occupations that involve directing and coordinating the efficient functioning of organizations, and require generating solutions and managing uncertainties were classified as 1. Sample job titles include directors and chief executives of major organizations, marketing and sales managers, and senior officers in fire, ambulance, prison and related services. Likewise, less creative occupations (i.e., conventional occupations) were coded as 0.	1, 0
Overall occupational creativity	UK SOC 2000 Job codes	Creative occupations, including artistic, scientific, and managerial occupations, were coded as 1. Detailed operationalization will be described below.<br>Less creative occupations (i.e., conventional occupations) were coded as 0. Conventional occupations are characterized by structured, orderly, and routine tasks that primarily involve working with things, numbers, or machines. Sample job titles include filing and other records assistants/clerks, counter clerks, and plastics process operatives.	1, 0
Creative achievement	US O*NET Database	1. Fluency of ideas: The ability to come up with a number of ideas about a topic (the number of ideas is important, not their quality, correctness, or creativity).<br>What level of fluency of ideas is needed to perform your current job?<br>2. Originality: The ability to come up with unusual or clever ideas about a given topic or situation, or to develop creative ways to solve a problem.<br>What level of originality is needed to perform your current job?<br>3. Thinking Creatively: Developing, designing, or creating new applications, ideas, relationships, systems, or products, including artistic contributions.<br>What level of thinking creatively is needed to perform your current job?	7-point scale: 1 = Lowest Level, 7 = Highest Level

Table S2. Summary of the creativity phenotypes in the UKB discovery sample by sex

Artistic creativity	N	Case (%)	Control (%)	Genomic control λ
Male	27,351	3896(14.24)	23455(85.76)	1.047
Female	37,229	2729(7.33)	34500(92.67)	1.047
Scientific creativity	N	Case (%)	Control (%)	Genomic control λ
Male	36,861	13406(36.37)	23455(63.63)	1.096
Female	39,319	4819(12.26)	34500(87.74)	1.096
Managerial creativity	N	Case (%)	Control (%)	Genomic control λ
Male	50,521	27066(53.57)	23455(46.43)	1.047
Female	50,432	15932(31.59)	34500(68.41)	1.047
Overall occupational creativity	N	Case (%)	Control (%)	Genomic control λ
Male	67,823	44368(65.42)	23455(34.58)	1.096
Female	57,980	23480(40.50)	34500(59.50)	1.096
Creative Achievement	N	Mean	SD	Genomic control λ
Male	108,684	3.33	0.81	1.147
Female	110789	3.16	0.76	1.199

Table S3. Summary of O*NET linked phenotype scores of creative achievement in the U.K. Biobank discovery data

	N	Mean (SD)
Creative achievement	219,473	3.24 (0.79)
Male with artistic creativity	3,896	4.21 (0.47)
Female with artistic creativity	2,729	4.22 (0.38)
Male with scientific creativity	12,490	4.26 (0.21)
Female with scientific creativity	4,403	4.15 (0.31)
Male with managerial creativity	23,667	2.87 (0.34)
Female with managerial creativity	13,245	3.75 (0.36)
Male with overall occupational creativity	40,053	4.02 (0.37)
Female with overall occupational creativity	20,377	3.90 (0.41)
Male with conventional occupations	21,319	2.25 (0.46)
Female with conventional occupations	30,020	2.23 (0.28)

Table S4. Phenotypic correlations among creativity phenotypes

		Artistic creativity	Scientific creativity	Managerial creativity	Overall occupational creativity
All	Creative achievement	.864***	.930***	.906***	.915***
Male	Creative achievement	.836***	.928***	.895***	.902***
Female	Creative achievement	.886***	.915***	.916***	.925***

Note. N = 25,215-139,912 *** p < .001

Table S5. Sample size and demographic distribution in replication samples

	UKB follow-up dataset			Add Health dataset			WLS dataset		
Artistic creativity	N=10,330			N=875			N=1,802		
Case (%)	695	6.73%		92	10.51%		116	10.51%	
Male (%)	371	53.38%		32	34.78%		57	49.14%	
Female (%)	324	46.62%		60	65.22%		59	50.86%	
Male (%)	3,380	32.72%		329	37.60%		645	35.79%	
Scientific creativity	N=14,305			N=856			N=1,809		
Case (%)	4,670	32.65%		73	8.53%		123	6.80%	
Male (%)	3,362	71.99%		43	58.90%		110	89.43%	
Female (%)	1,308	28.01%		30	41.10%		13	10.57%	
Male (%)	6,371	44.54%		340	39.72%		698	38.58%	
Managerial creativity	N=17,884			N=1,296			N=2,026		
Case (%)	8,249	46.13%		513	39.58%		340	16.78%	
Male (%)	4,901	59.41%		258	50.29%		199	58.53%	
Female (%)	3,348	40.59%		255	49.71%		141	41.47%	
Male (%)	7,910	44.23%		555	42.82%		787	38.85%	
Overall occupational creativity	N=23,249			N=1,461			N=2,265		
Case (%)	13,614	58.56%		678	46.41%		579	25.56%	
Male (%)	8,634	63.42%		333	49.12%		366	63.21%	
Female (%)	4,980	36.58%		345	50.88%		213	36.79%	
Male (%)	11,643	50.08%		630	43.12%		954	42.12%	
	N	Mean (SD)	Range	N	Mean (SD)	Range	N	Mean (SD)	Range
Creative achievement	30,837	3.403(0.772)	[1.027, 4.743]	3,711	3.185(0.735)	[1.027, 4.763]	5,431	3.049(0.647)	[1.227, 4.854]
Male	13,396	3.567(0.767)	[1.027, 4.743]	1,627	3.187(0.756)	[1.027, 4.757]	2,653	3.128(0.683)	[1.227, 4.854]
Female	17,441	3.278(0.753)	[1.027, 4.743]	2,084	3.183(0.718)	[1.307, 4.763]	2,778	2.974(0.600)	[1.307, 4.452]

Note. Artistic creativity, scientific creativity, managerial creativity, and Overall occupational creativity were dichotomous variables; creative achievement was a continuous variable.

Table S6. Genomic control λ for GWAS on creativity phenotypes in UKB data

	Phenotype	Genomic control λ	LDSC Intercept (S.E.)
All	artistic creativity	1.147	1.011 (0.0066)
	scientific creativity	1.147	1.012 (0.0080)
	managerial creativity	1.096	1.016 (0.0069)
	Overall occupational creativity	1.147	1.025 (0.0074)
	creative achievement	1.310	1.049 (0.0093)
Male	artistic creativity	1.047	0.994 (0.0065)
	scientific creativity	1.096	1.009 (0.0075)
	managerial creativity	1.047	1.018 (0.0068)
	Overall occupational creativity	1.096	1.016 (0.0072)
	creative achievement	1.147	1.027 (0.0077)
Female	artistic creativity	1.047	1.010 (0.0062)
	scientific creativity	1.096	0.999 (0.0066)
	managerial creativity	1.047	1.003 (0.0063)
	Overall occupational creativity	1.096	1.004 (0.0101)
	creative achievement	1.199	1.021 (0.0074)

Note. LDSC intercept - Intercepts of univariate linkage disequilibrium score regression

Table S7-1. Summary of top loci for creativity traits identified from UKB discovery sample

Locus	Phenotypes	SNP	CHR	BP	Function	Nearest genes	A1/A2	Allele Freq	β (SE)	*P-discovery*	*p-value in Meta-analysis*
$P < 1.0 \times 10^{-8}$											
1	Scientific creativity	rs2613492	1	72758984	intergenic	*NEGR1; LINC01360*	C/T	0.2747	-0.0136(0.0024)	2.20E-08	2.47×10^{-9}
	Overall occupational creativity	rs2613492	1	72758984	intergenic		C/T	0.2729	-0.0130(0.0022)	6.70E-09	3.16×10^{-10}
	Creative achievement	rs12130762	1	72153088	intronic		G/A	0.9009	-0.0234(0.0041)	8.60E-09	7.11×10^{-10}
2	Creative achievement	rs2880052	2	44768265	intronic	*CAMKMT*	G/A	0.3325	0.0158(0.0025)	2.30E-10	2.17×10^{-9}
3	Creative achievement	rs7581162	2	60704484	intronic	*BCL11A*	T/A	0.5666	0.0142(0.0024)	2.70E-09	1.60×10^{-10}
4	Creative achievement	rs11678979	2	100802891	intergenic	*AFF3; LINC01104*	T/C	0.7297	-0.0185(0.0027)	4.10E-12	6.79×10^{-12}
5	Creative achievement	rs7601502	2	212617805	intronic	*ERBB4*	G/A	0.7008	0.0152(0.0026)	3.10E-09	7.23×10^{-10}
6	Scientific creativity	rs533904333 (new id rs71077794)	3	49385350	intergenic	*GPX1; USP4; NICN1; DAG1; MON1A*	A/AT	0.7275	-0.0170(0.0025)	5.70E-12	5.17×10^{-13}
	Overall occupational creativity	rs71324962	3	49487487	intergenic		A/C	0.6948	-0.0127(0.0021)	6.70E-10	5.72×10^{-11}
	Creative achievement	rs34034116	3	49959156	intronic		C/A	0.5696	0.0195(0.0024)	2.10E-16	2.64×10^{-18}
7	Scientific creativity	rs72757186	5	60246469	intronic	*NDUFAF2; PART1; ZSWIM6*	T/G	0.7753	-0.0162(0.0026)	6.10E-10	8.58×10^{-11} (rs7715147)
	Overall occupational creativity	rs26950	5	59823118	intronic		/				1.75×10^{-10}
	Creative achievement	rs10223052	5	60800336	intronic		A/G	0.3554	0.0171(0.0025)	4.50E-12	6.32×10^{-13}
8*	Creative achievement	rs56194430	5	67824690	intergenic	*PIK3R1; LINC02198*	C/T	0.8301	0.0187(0.0032)	3.10E-09	1.43×10^{-8}
9	Managerial creativity	rs1487441	6	98553894	intergenic	*MIR2113;PNKY*	G/A	0.5170	-0.0121(0.0021)	1.40E-08	2.50×10^{-8}
	Scientific creativity	rs9387963	6	98423208	intergenic		G/A	0.4415	0.0111(0.0021)	1.20E-07	5.76×10^{-9}
	Overall occupational creativity	rs1487441	6	98553894	intergenic		G/A	0.5149	-0.0135(0.0019)	1.40E-12	1.62×10^{-12}
	Creative achievement	rs1487441	6	98553894	intergenic		G/A	0.5152	-0.0212(0.0024)	3.10E-19	3.70×10^{-23}
10	Creative achievement	rs11793831	9	23362311	intergenic	*LINC01239; LOC101929563*	G/T	0.5841	-0.0163(0.0024)	1.00E-11	4.26×10^{-11}
11	Creative achievement	rs68119843	9	124604538	intronic	*TTLL11*	A/T	0.4447	-0.0148(0.0024)	5.70E-10	4.80×10^{-12}
12	Creative achievement	rs9537814	13	58371672	intergenic	*PCDH17; LINC02338*	T/G	0.7191	0.0154(0.0026)	4.50E-09	9.09×10^{-10}
13	Creative achievement	rs772661599 (new id rs200794414)	14	23441543	UTR3	*AJUBA*	A/AAAGT	0.5983	0.0140(0.0024)	8.40E-09	2.06×10^{-9} (rs59841088)
14*	Creative achievement	rs7171500	15	56988732	intronic	*ZNF280D*	T/A	0.8036	-0.0176(0.003)	3.20E-09	2.40×10^{-7}
15*	Scientific creativity	rs7200666	16	55121234	intergenic	*IRX5; IRX6*	T/C	0.9843	-0.0495(0.0084)	4.60E-09	3.63×10^{-8}
16*	Creative achievement	rs117404634	17	19819469	intronic	*AKAP10*	G/A	0.9872	0.0631(0.0109)	8.20E-09	7.97×10^{-6}
17*	Overall occupational creativity	rs2732645	17	44254291	intronic	*KANSL1*	C/T	0.8346	0.0161(0.0028)	8.30E-09	2.46×10^{-6} (rs2696569)
18*	Creative achievement	rs5821228	17	56459271	intronic	*RNF43*	G/GA	0.6038	0.0135(0.0024)	2.20E-08	7.15×10^{-8}
	Overall occupational creativity	17:57069683_AAT_A	17	57069683	intronic	*TRIM37*	AAT/A	0.3706	-0.0117(0.002)	3.90E-09	1.66×10^{-7}

19	Artistic creativity	rs4800011	18	35798082	intergenic	*MIR4318; CELF4*	A/G	0.5306	0.0090(0.0017)	6.40E-08	1.69×10^{-8} (rs5824114)
	Overall occupational creativity	rs11877758	18	35138110	intronic		T/G	0.6883	0.0126(0.0021)	1.10E-09	1.89×10^{-9}
	Creative achievement	rs9964724	18	35159124	intergenic		C/T	0.3182	-0.0163(0.0025)	1.20E-10	2.07×10^{-10}
$P < 5.0 \times 10^{-8}$											
1*	Creative achievement	rs17379561	1	98340139	intronic	*DPYD*	A/T	0.8562	0.0186(0.0033)	2.50E-08	1.61×10^{-7}
2*	Overall occupational creativity	rs4440360	5	12243796	intergenic	*CTNND2; LINC01194*	A/C	0.6206	0.0107(0.002)	4.30E-08	6.66×10^{-8}
3	Creative achievement	rs448809	5	88005828	ncRNA_intronic	*MEF2C-AS2*	G/T	0.4178	0.0151(0.0024)	3.60E-10	4.35×10^{-12}
4	Creative achievement	6:31551303_ACT_A	6	31551303	intergenic	*LTB; LST1*	ACT/A	0.3868	0.0136(0.0024)	2.20E-08	2.00×10^{-9}
5*	Creative achievement	rs12204714	6	152235339	intronic	*ESR1*	C/T	0.3687	-0.0136(0.0024)	2.30E-08	1.35×10^{-8}
6	Creative achievement	rs2240857	7	8010634	intronic	*GLCCI1*	T/G	0.8579	0.0191(0.0034)	1.90E-08	6.04×10^{-9}
7*	Creative achievement	10:63574533_TA_T	10	63574533	intergenic	*CABCOCO1; ARID5B*	TA/T	0.4690	-0.0138(0.0025)	3.60E-08	5.91×10^{-8}
8	Creative achievement	rs9783478	12	82344954	intergenic	*PPFIA2; LINC02426*	A/G	0.6738	-0.0142(0.0025)	1.50E-08	4.54×10^{-9}
9*	Artistic creativity	15:90776161_CA_C	15	90776161	intronic	*CIB1*	CA/C	0.7574	-0.0108(0.002)	5.00E-08	8.47×10^{-7}
10	Creative achievement	rs531139717	16	28572281	intronic	*SGF29*	C/CA	0.6637	0.0142(0.0025)	2.10E-08	1.22×10^{-9}
11*	Creative achievement	rs1108343	16	51211595	intergenic	*SALL1; LINC01571*	T/C	0.3514	-0.0136(0.0025)	3.30E-08	4.27×10^{-8}
12	Creative achievement	rs62098013	18	50863861	intronic	*DCC*	G/A	0.6381	0.0138(0.0025)	2.50E-08	3.88×10^{-10}

Note. A1, effect allele; A2, reference allele; Allele Freq, the minor allele frequency; SE, standard error; *P-het*, heterogeneity *p*-value across discovery and replication samples.

Meta-analysis sample included individuals of European ancestry from the discovery UKB dataset, UKB follow-up cohorts, the Add Health and WLS dataset.

For the variant not available in the meta-analysis results, proxy SNP with r^>0.9 was selected. * loci not reaching genome-wide significant at 1×10^{-8} for either creativity traits in the meta-analysis.

Table S7-2. A meta-analysis of top loci for creativity variables across UKB, Add Health, and WLS datasets

Locus	Phenotypes	SNP	CHR	BP	Function	Nearest genes	A1/A2	Allele Freq	Effect Size	SE	*P_meta*	*P_het*
1	Scientific creativity	rs2613492	1	72758984	intergenic	*NEGR1; LINC01360*	C/T	0.2747	-0.0711	0.0119	2.47×10^{-9}	0.2022
	Overall occupational creativity	rs2613492	1	72758984	intergenic		C/T	0.2729	-0.0516	0.0082	3.16×10^{-10}	0.3991
	Creative achievement	rs12130762	1	72153088	intronic		G/A	0.9009	-0.0229	0.0037	7.11×10^{-10}	0.3511
2	Creative achievement	rs2880052	2	44768265	intronic	*CAMKMT*	G/A	0.3325	0.0136	0.0023	2.17×10^{-9}	0.1736
3	Creative achievement	rs7581162	2	60704484	intronic	*BCL11A*	T/A	0.5666	0.0139	0.0022	1.60×10^{-10}	0.8697
4	Creative achievement	rs11678979	2	100802891	intergenic	*AFF3; LINC01104*	T/C	0.7297	-0.0167	0.0024	6.79×10^{-12}	0.366
5	Creative achievement	rs7601502	2	212617805	intronic	*ERBB4*	G/A	0.7008	0.0144	0.0023	7.23×10^{-10}	0.3326
6	Scientific creativity	rs533904333 (new id rs71077794)	3	49385350	intergenic	*GPX1; USP4; NICN1;DAG1; MON1A*	A/AT	0.7275	-0.0876	0.0121	5.17×10^{-13}	0.1604
	Overall occupational creativity	rs71324962	3	49487487	intergenic		A/C	0.6948	-0.0495	0.0076	5.72×10^{-11}	0.8792
	Creative achievement	rs34034116	3	49959156	intronic		C/A	0.5696	0.0189	0.0022	2.64×10^{-18}	0.507
7	Scientific creativity	rs7715147	5	60053220	intronic	*NDUFAF2; PART1; ZSWIM6*	A/C	0.2420	0.0782	0.0121	8.58×10^{-11}	0.0737
	Overall occupational creativity	rs26950	5	59823118	intronic		T/C	0.4038	0.0454	0.0071	1.75×10^{-10}	0.1318
	Creative achievement	rs10223052	5	60800336	intronic		A/G	0.3554	0.0164	0.0023	6.32×10^{-13}	0.7715
8	Creative achievement	rs448809	5	88005828	ncRNA_intronic	*MEF2C-AS2*	G/T	0.4178	0.0152	0.0022	4.35×10^{-12}	0.6992
9	Creative achievement	6:31551303_ACT_A	6	31551303	intergenic	*LTB; LST1*	ACT/A	0.3868	0.0137	0.0023	2.00×10^{-9}	0.9892
10	Managerial creativity	rs1487441	6	98553894	intergenic	*MIR2113; PNKY*	G/A	0.5170	-0.0443	0.0079	2.50×10^{-8}	0.3665
	Scientific creativity	rs9387963	6	98423208	intergenic		G/A	0.4415	0.0596	0.0102	5.76×10^{-9}	0.927
	Overall occupational creativity	rs1487441	6	98553894	intergenic		G/A	0.5149	-0.0494	0.0070	1.62×10^{-12}	0.2363
	Creative achievement	rs1487441	6	98553894	intergenic		G/A	0.5152	-0.0213	0.0022	3.70×10^{-23}	0.9442
11	Creative achievement	rs2240857	7	8010634	intronic	*GLCCI1*	T/G	0.8579	0.0180	0.0031	6.04×10^{-9}	0.5147
12	Creative achievement	rs11793831	9	23362311	intergenic	*LINC01239; LOC101929563*	G/T	0.5841	-0.0144	0.0022	4.26×10^{-11}	0.2777
13	Creative achievement	rs68119843	9	124604538	intronic	*TTLL11*	A/T	0.4447	-0.0151	0.0022	4.80×10^{-12}	0.8541
14	Creative achievement	rs9783478	12	82344954	intergenic	*PPFIA2; LINC02426*	A/G	0.6738	-0.0134	0.0023	4.54×10^{-9}	0.7034
15	Creative achievement	rs9537814	13	58371672	intergenic	*PCDH17; LINC02338*	T/G	0.7191	0.0147	0.0024	9.09×10^{-10}	0.3492
16	Creative achievement	rs59841088	14	23440049	UTR3	*AJUBA*	A/G	0.3983	-0.0132	0.0022	2.06×10^{-9}	0.8429
17	Creative achievement	rs531139717	16	28572281	intronic	*SGF29*	C/CA	0.6637	0.0140	0.0023	1.22×10^{-9}	0.6809
18	Artistic creativity	rs5824114	18	35800866		*MIR4318; CELF4*	A/AC	0.5176	0.0960	0.0170	1.69×10^{-8}	0.6515
	Overall occupational creativity	rs11877758	18	35138110	intronic		T/G	0.6883	0.0455	0.0076	1.89×10^{-9}	0.2045
	Creative achievement	rs9964724	18	35159124	intergenic		C/T	0.3182	-0.0147	0.0023	2.07×10^{-10}	0.1266
19	Creative achievement	rs62098013	18	50863861	intronic	*DCC*	G/A	0.6381	0.0141	0.0023	3.88×10^{-10}	0.8995

Lead variants shown were *P-meta* $< 5 \times 10^{-8}$ for artistic creativity, scientific creativity, managerial creativity, overall occupational creativity, and creative achievement in individuals of European ancestry from the meta-analysis of GWAS for discovery UKB dataset, UKB follow-up cohorts, the Add Health and WLS dataset.

For artistic creativity, the sample size for the U.K. Biobank discovery: N= 64,580; All: N = 77,587. For scientific creativity, the sample size for the U.K. Biobank discovery: N= 76,180; All: N = 93,150. For managerial creativity, the sample size for the U.K. Biobank discovery: N= 100,953; All: N = 122,159. For overall occupational creativity, the sample size for the U.K. Biobank discovery: N= 125,803; All: N = 152,778. For creative achievement, the sample size for the U.K. Biobank discovery: N = 219,473; All: N = 259,452. The replication studies include the UKB follow-up cohort, Add Health study, and Wisconsin Longitudinal study. All significant variants from the U.K. Biobank discovery samples were presented in Supplementary Table S6.

SNP, single-nucleotide polymorphism; CHR, chromosome; A1, effect allele; A2, reference allele; Allele Freq, the minor allele frequency; SE, standard error; *P-het*, heterogeneity *p*-value across discovery and replication samples.

Table S8. A meta-analysis of top loci for creativity variables across UKB, AddHealth, and WLS datasets by sex

Locus	Phenotypes	SNP	CHR	BP	Function	Nearest genes	A1/A2	Allele Freq	Effect Size	SE	*P_meta*	*P_het*
Male												
1	Creative achievement	2:10936626_TA_T	2	10936626	intronic	*PDIA6*	TA/T	0.8786	-0.0243	0.0052	2.84E-06	0.0006
2	Creative achievement	rs756277696	2	58719459	intergenic	*LINC01795; LINC01122*	CTCCT/C	0.8324	-0.0223	0.0044	3.15E-07	0.0054
3	Creative achievement	rs11691869	2	100805996	intergenic	*AFF3; LINC01104*	C/A	0.6374	-0.0221	0.0034	4.59E-11	0.9030
4	Creative achievement	rs773674547	3	49428822	intronic	*RHOA*	CA/C	0.6902	-0.0203	0.0035	8.78E-09	0.0856
5	Creative achievement	rs72757186	5	60246469	intronic	*NDUFAF2*	T/G	0.7752	-0.0237	0.0041	6.95E-09	0.1524
6	Creative achievement	rs1487441	6	98553894	intergenic	*MIR2113; PNKY*	G/A	0.5156	-0.0217	0.0032	1.03E-11	0.8234
7	Creative achievement	rs7467480	9	23354940	intergenic	*LINC01239; LOC101929563*	T/A	0.5861	-0.0156	0.0032	1.27E-06	0.0522
8	Scientific creativity	rs145920985	10	67946039	intronic	*CTNNA3*	A/AGTGTGT	0.6106	0.0771	0.0144	8.92E-08	0.0633
9	Creative achievement	16:51187247_CTT_C	16	51187247	intergenic	*SALL1; LINC01571*	CTT/C	0.7984	0.0241	0.0041	2.95E-09	0.1284
Female												
1	Scientific creativity	rs6656590	1	214751786	intergenic	*PTPN14; CENPF*	T/C	0.3301	-0.0994	0.0204	1.04E-06	0.0397
2	Creative achievement	3:49959570_CA_C	3	49959570	intronic	*MON1A*	CA/C	0.4765	0.0221	0.0032	8.67E-12	1.0000
3	Scientific creativity	rs9847000	3	94067218	intergenic	*NSUN3; MIR6730*	A/G	0.7095	0.1227	0.0209	4.59E-09	0.0320
4	Creative achievement	rs10223052	5	60800336	intronic	*ZSWIM6*	A/G	0.3557	0.0193	0.0033	6.52E-09	0.4794
5	Creative achievement	rs151077586	6	66616066	intergenic	*SLC25A51P1; NONE*	C/CA	0.7781	0.0216	0.0039	2.81E-08	1.0000
6	Overall occupational creativity	rs1487441	6	98553894	intergenic	*MIR2113; PNKY*	G/A	0.5142	-0.0616	0.0107	8.47E-09	0.0651
	Creative achievement	rs1487441	6	98553894	intergenic	*MIR2113; PNKY*	G/A	0.5148	-0.0218	0.0031	2.82E-12	0.8974
7	Creative achievement	rs1914391	7	71690601	intronic	*CALN1*	C/T	0.5562	0.0147	0.0029	4.83E-07	0.0441
8	Scientific creativity	rs117261335	9	128503542	intergenic	*MAPKAP1; LOC51145*	C/T	0.9439	-0.2582	0.0423	1.07E-09	0.5267
9	Creative achievement	rs890547	18	35204857	intergenic	*CELF4; MIR4318*	G/T	0.3647	-0.0167	0.0030	4.14E-08	0.0194

Note. A1, effect allele; A2, reference allele; Allele Freq, the minor allele frequency; SE, standard error; *P-het*, heterogeneity *p*-value across discovery and replication samples.

Table S9 Summary of common SNP heritability estimations for creativity phenotypes from GWAS results in UKB data

	Artistic creativity	Scientific creativity	Managerial creativity	Overall occupational creativity	Creative achievement
Liability-scale h^2 (95% CI)	0.2152 [0.18, 0.25]	0.1845 [0.16, 0.21]	0.0787 [0.06, 0.09]	0.1218 [0.11, 0.14]	
Observed-scale h^2 (95% CI)	0.1103 [0.09, 0.13]	0.1398 [0.12, 0.16]	0.0618 [0.05, 0.07]	0.0848 [0.07, 0.10]	0.0939 [0.09, 0.10]
	Artistic VS scientific creativity	Artistic VS managerial creativity	Scientific VS managerial creativity		
Comparison of *Liability-scale* h^2 (*p* value)	1.4552 (0.15)	7.7873 (6.84×10^{-15})	7.1792 (7.01×10^{-13})		

Table S10. Genetic correlations for creativity phenotypes across sex in the UKB sample

Overall Genetic Correlation (S.E.)	Artistic creativity	Scientific creativity	Managerial creativity	Overall occupational creativity	
Scientific creativity	0.7691 (0.0403)				
Managerial creativity	0.6395 (0.0506)	0.6603 (0.0344)			
Overall occupational creativity	0.8205 (0.031)	0.8809 (0.0148)	0.9271 (0.0081)		
Creative achievement	0.8235 (0.028)	0.9235 (0.0156)	0.8347 (0.0223)	0.9608 (0.0108)	
	**Artistic creativity**	**Scientific creativity**	**Managerial creativity**	**Overall occupational creativity**	**Creative achievement**
Genetic Correlation Across Sexes (S.E.)	0.8724 (0.1063)	0.8000 (0.0693)	0.8141 (0.1124)	0.8892 (0.0993)	0.8838 (0.0379)

Table S11. Sources and phenotype descriptions for GWAS results used in the genetic correlation analysis.

Phenotypes	PMID	Sample size	Phenotype coding
**Personal Traits**			
Agreeableness	21173776	20669 (17375 discovery samples + 3294 replication samples)	continuous variable
Conscientiousness	21173776	20669 (17375 discovery samples + 3294 replication samples)	continuous variable
Extraversion	21173776	20669 (17375 discovery samples + 3294 replication samples)	continuous variable
Openness to experience	21173776	20669 (17375 discovery samples + 3294 replication samples)	continuous variable
Neuroticism	29942085	449484	continuous variable (weighted sum-score of 12 dichotomous items and sum-score of five-point Likert scale with 8 items and 12 items, respectively)
Risk tolerance	30643258	466571	binary variable
Intelligence	5665562	269867	continuous variable and binary variable
Height	34140656	697242	continuous variable
Educational achievement	30038396	766345	continuous variable (years of education)
**Health and Well-Being**			
ADHD	30478444	19,099 cases/34,194 controls	binary variable
Alcohol misuse	34140656	N=131660, GWASeqN=141521	ordinal
Autism	30804558	18,381 cases/27,969 controls	binary variable
Schizophrenia	25056061	36989cases/113075controls	binary variable
Bipolar disorder	31043756	20,352 cases/31,358 controls	binary variable
BMI	34140656	672434	continuous variable
Number of cancer illness	UKB data-field 134	361136	integer (0,1,2,3 and4)
Number of non-cancer illness	UKB data-field 135	361141	integer (0,1 and 2)
Subjective wellbeing	27089181	298420	continuous variable
Longevity	31413261	11,262 cases/25,483 controls	binary variable (cases were defined as individuals who survive at the age corresponding to the 90th survival percentile, and controls are those whose age at death or at last contact was at or below the age corresponding to the 60th survival percentile)
Cannabis use	34140656	179409	integer
NSEX (Number of sexual partners)	34446935	336121	integer
FSEX (Age first had sexual intercourse)	UKB data-field 2139	317694	integer
**Career success**			
Income	31844048	286301	integer (5-point scale corresponding to the total household income before tax)
Job satisfaction	UKB data-field 4537	82190	ordinal
Occupational status	O*NET data	219483	continuous variable: an overall composite index of levels of abilities, skills, and knowledge
Number ever born (men)	34140656	295449	integer
Number ever born (women)	34140656	453439	integer
Age first birth	34140656	438596	integer

Table S12. Genetic correlations between creativity and outcome variables

Phenotypes	Artistic creativity						Scientific creativity						Managerial creativity						Overall occupational creativity						Creative achievement					
			95% CI						95% CI						95% CI						95% CI						95% CI			
	r_g	S.E.	Lower	Upper	*P*	FDR	r_g	S.E.	Lower	Upper	*P*	FDR	r_g	S.E.	Lower	Upper	*P*	FDR	r_g	S.E.	Lower	Upper	*P*	FDR	r_g	S.E.	Lower	Upper	*P*	FDR
Personal Traits																														
Agreeableness	0.120	0.314	-0.495	0.735	0.7028		0.810	0.883	-0.920	2.541	0.3586		-0.002	0.285	-0.561	0.557	0.9948		0.312	0.400	-0.472	1.096	0.4356		0.472	0.526	-0.559	1.503	0.3694	
Conscientiousness	-0.156	0.130	-0.410	0.098	0.2286		0.075	0.113	-0.147	0.297	0.5098		0.182	0.149	-0.110	0.473	0.2229		0.089	0.119	-0.144	0.321	0.4565		0.026	0.098	-0.166	0.218	0.7898	
Extraversion	0.066	0.161	-0.250	0.381	0.6842		0.201	0.144	-0.081	0.482	0.163		0.504	0.213	0.087	0.921	0.0178	<0.05	0.365	0.165	0.042	0.687	0.0266	<0.05	0.240	0.130	-0.014	0.494	0.0641	
Openness	0.636	0.138	0.366	0.906	3.96E-06	<0.05	0.558	0.104	0.356	0.761	6.91E-08	<0.05	0.303	0.122	0.065	0.541	0.0126	<0.05	0.507	0.107	0.298	0.716	1.99E-06	<0.05	0.520	0.093	0.338	0.702	2.15E-08	<0.05
Neuroticism	-0.104	0.034	-0.171	-0.037	0.0023	<0.05	-0.211	0.029	-0.268	-0.154	5.13E-13	<0.05	-0.248	0.035	-0.316	-0.180	7.51E-13	<0.05	-0.235	0.029	-0.291	-0.179	2.51E-16	<0.05	-0.231	0.024	-0.279	-0.184	2.07E-21	<0.05
Risk tolerance	0.119	0.036	0.048	0.190	0.0010	<0.05	0.020	0.035	-0.048	0.089	5.63E-01		0.361	0.039	0.285	0.437	1.01E-20	<0.05	0.244	0.033	0.178	0.309	2.34E-13	<0.05	0.093	0.026	0.042	0.144	3.00E-04	<0.05
Intelligence	0.460	0.038	0.387	0.534	1.38E-34	<0.05	0.682	0.030	0.624	0.741	1.87E-115	<0.05	0.462	0.041	0.382	0.542	1.93E-29	<0.05	0.588	0.030	0.528	0.647	3.25E-84	<0.05	0.677	0.020	0.637	0.717	4.24E-241	<0.05
Height	0.147	0.027	0.095	0.200	3.01E-08	<0.05	0.168	0.025	0.120	0.216	6.74E-12	<0.05	0.198	0.029	0.142	0.253	4.09E-12	<0.05	0.201	0.024	0.155	0.247	1.13E-17	<0.05	0.209	0.018	0.175	0.243	1.03E-32	<0.05
Educational achievement	0.737	0.030	0.678	0.796	1.11E-130	<0.05	0.928	0.025	0.880	0.977	1.01E-310	<0.05	0.683	0.030	0.624	0.742	3.82E-114	<0.05	0.863	0.022	0.820	0.905	0.0000	<0.05	0.936	0.013	0.910	0.962	0.0000	<0.05
Health and Well-Being																														
ADHD	-0.302	0.053	-0.406	-0.197	1.46E-08	<0.05	-0.430	0.042	-0.512	-0.347	1.96E-24	<0.05	-0.189	0.051	-0.289	-0.089	0.0002	<0.05	-0.309	0.042	-0.391	-0.227	1.63E-13	<0.05	-0.429	0.034	-0.496	-0.362	5.39E-36	<0.05
Alcohol misuse	0.050	0.039	-0.027	0.126	0.2064		0.046	0.038	-0.028	0.121	0.2242		0.125	0.055	0.018	0.231	0.0223	<0.05	0.097	0.043	0.013	0.180	0.0236	<0.05	0.105	0.033	0.040	0.170	0.0015	<0.05
Autism	0.156	0.056	0.047	0.265	0.0051	<0.05	0.286	0.052	0.185	0.388	3.14E-08	<0.05	0.038	0.064	-0.088	0.164	0.5545		0.161	0.050	0.062	0.259	0.0014	<0.05	0.149	0.044	0.064	0.234	0.0006	<0.05
Schizophrenia	0.165	0.034	0.098	0.233	1.54E-06	<0.05	0.088	0.029	0.031	0.146	0.0026	<0.05	0.010	0.038	-0.065	0.085	0.7985		0.075	0.030	0.016	0.133	0.0122	<0.05	0.057	0.025	0.008	0.106	0.0217	<0.05
Bipolar disorder	0.248	0.042	0.165	0.330	3.33E-09	<0.05	0.123	0.036	0.052	0.193	6.00E-04	<0.05	0.191	0.047	0.099	0.282	4.35E-05	<0.05	0.202	0.038	0.128	0.276	9.45E-08	<0.05	0.193	0.031	0.133	0.253	3.11E-10	<0.05
BMI	-0.352	0.027	-0.404	-0.300	1.35E-39	<0.05	-0.325	0.026	-0.377	-0.274	8.01E-35	<0.05	-0.148	0.032	-0.212	-0.084	4.77E-06	<0.05	-0.262	0.028	-0.316	-0.208	1.73E-21	<0.05	-0.267	0.022	-0.310	-0.224	2.69E-34	<0.05
Cancer illness	-0.022	0.081	-0.182	0.137	0.7845		-0.093	0.068	-0.225	0.040	0.1701		-0.028	0.105	-0.233	0.177	0.7897		-0.034	0.079	-0.189	0.121	0.6694		-0.022	0.060	-0.140	0.096	0.7151	
Non-cancer illness	-0.287	0.037	-0.359	-0.215	5.35E-15	<0.05	-0.304	0.036	-0.373	-0.234	1.37E-17	<0.05	-0.202	0.041	-0.282	-0.121	9.06E-07	<0.05	-0.276	0.035	-0.345	-0.207	3.86E-15	<0.05	-0.313	0.026	-0.365	-0.262	1.36E-32	<0.05
Subjective wellbeing	-0.035	0.053	-0.139	0.069	0.5077		0.058	0.048	-0.036	0.151	0.2261		0.205	0.062	0.084	0.326	0.0009	<0.05	0.128	0.047	0.036	0.220	0.0063	<0.05	0.133	0.036	0.061	0.204	0.0003	<0.05
Longevity	0.193	0.084	0.028	0.358	0.0219	<0.05	0.162	0.072	0.021	0.302	0.0245	<0.05	-0.036	0.082	-0.197	0.126	0.6666		0.076	0.072	-0.066	0.218	0.294		0.156	0.060	0.039	0.272	0.0091	<0.05
Cannabis Use	0.450	0.046	0.360	0.540	1.43E-22	<0.05	0.345	0.046	0.255	0.435	6.61E-14	<0.05	0.292	0.050	0.194	0.390	5.70E-09	<0.05	0.376	0.042	0.294	0.459	3.01E-19	<0.05	0.374	0.035	0.305	0.443	2.83E-26	<0.05
Nsex	0.118	0.033	0.055	0.182	0.0003	<0.05	-0.063	0.032	-0.125	-0.001	4.77E-02		0.170	0.035	0.102	0.238	7.97E-07	<0.05	0.104	0.030	0.046	0.162	4.00E-04	<0.05	0.001	0.025	-0.048	0.050	9.69E-01	
FSEX	0.427	0.030	0.368	0.485	6.91E-46	<0.05	0.593	0.025	0.543	0.642	4.99E-122	<0.05	0.242	0.033	0.177	0.308	3.94E-13	<0.05	0.428	0.024	0.380	0.475	1.17E-70	<0.05	0.559	0.020	0.521	0.597	3.30E-181	<0.05
Career Success																														
Income	0.584	0.037	0.512	0.655	1.31E-57	<0.05	0.747	0.030	0.689	0.805	7.45E-142	<0.05	0.830	0.035	0.762	0.899	8.67E-124	<0.05	0.844	0.025	0.794	0.894	1.12E-242	<0.05	0.873	0.019	0.836	0.910	0.0000	<0.05
Job satisfaction	0.094	0.082	-0.068	0.255	0.2549		0.049	0.061	-0.071	0.169	0.4202		0.208	0.080	0.052	0.364	0.009	<0.05	0.138	0.067	0.006	0.269	0.0404	<0.05	0.084	0.054	-0.022	0.191	0.1199	
Occupational status	0.643	0.045	0.554	0.732	9.63E-46	<0.05	0.870	0.029	0.814	0.927	2.88E-201	<0.05	0.788	0.033	0.722	0.853	1.18E-122	<0.05	0.887	0.023	0.842	0.932	0.0000	<0.05	0.898	0.014	0.871	0.924	0.0000	<0.05
Number ever born (men)	-0.238	0.057	-0.349	-0.126	2.89E-05	<0.05	-0.346	0.055	-0.454	-0.237	4.10E-10	<0.05	-0.082	0.073	-0.224	0.060	0.2594		-0.220	0.058	-0.333	-0.107	0.0001	<0.05	-0.276	0.046	-0.365	-0.187	1.37E-09	<0.05
Number ever born (women)	-0.346	0.058	-0.459	-0.233	1.79E-09	<0.05	-0.354	0.048	-0.449	-0.259	2.74E-13	<0.05	-0.144	0.066	-0.272	-0.016	0.0277	<0.05	-0.335	0.064	-0.460	-0.211	1.37E-07	<0.05	-0.298	0.039	-0.374	-0.222	1.76E-14	<0.05
Age first birth	0.539	0.035	0.470	0.608	3.59E-53	<0.05	0.687	0.029	0.630	0.745	1.49E-122	<0.05	0.458	0.035	0.390	0.526	1.49E-39	<0.05	0.611	0.027	0.559	0.663	4.54E-117	<0.05	0.691	0.020	0.651	0.731	3.83E-252	<0.05

Table S13. Comparison of genetic correlations between creativity phenotypes and outcome variables

	Artistic creativity vs. Scientific creativity	Artistic creativity vs. Managerial creativity	Scientific creativity vs. Managerial creativity
**Personal Traits**			
Agreeableness	0.4610	0.7744	0.3813
Conscientiousness	0.1800	0.0872	0.5681
Extraversion	0.5317	0.1002	0.2369
Openness to experience	0.6534	0.0701	0.1097
Neuroticism	0.0167	0.0029	0.4138
Risk tolerance	0.0497	4.77E-06	6.81E-11
Intelligence	3.72E-06	0.9756	1.43E-05
Height	0.5671	0.1979	0.4325
Educational achievement	9.27E-07	0.2078	2.79E-10
**Health and Well-Being**			
ADHD	0.0601	0.1263	0.0003
Alcohol misuse	0.9518	0.2639	0.2386
Autism	0.0854	0.1654	0.0026
Schizophrenia	0.0884	0.0025	0.1034
Bipolar disorder	0.0238	0.3670	0.2476
BMI	0.4787	1.18E-06	2.19E-05
No of cancer illness	0.5056	0.9663	0.6024
No of non-cancer illness	0.7440	0.1212	0.0604
Subjective wellbeing	0.1931	0.0032	0.0588
Longevity	0.7778	0.0525	0.0713
Cannabis use	0.1069	0.0202	0.4350
NSEX (Number of sexual partners)	6.64E-05	0.2754	6.74E-07
FSEX (Age first had sexual intercourse)	2.22E-05	4.00E-05	0.0000
**Career success**			
Income	0.0005	1.12E-06	0.0693
Job satisfaction	0.6642	0.3180	0.1136
Occupational status	2.25E-05	0.0101	0.0608
Number ever born (men)	0.1731	0.0903	0.0038
Number ever born (women)	0.9163	0.0206	0.0100
Age first birth	0.0012	0.1004	4.42E-07

Note. Bonferroni-adjusted p-values cutoff is 0.00152

Table S14. Genetic correlations between creativity and outcome variables, partialling out intelligence

**Phenotypes**	**Artistic creativity**						**Scientific creativity**						**Managerial creativity**						**Overall occupational creativity**						**Creative achievement**					
			95% CI						95% CI						95% CI						95% CI						95% CI			
	r_g	S.E.	Lower	Upper	*P*	FDR	r_g	S.E.	Lower	Upper	*P*	FDR	r_g	S.E.	Lower	Upper	*P*	FDR	r_g	S.E.	Lower	Upper	*P*	FDR	r_g	S.E.	Lower	Upper	*P*	FDR
Personal Traits																														
Agreeableness	-0.144	0.282	-0.696	0.408	0.6097		0.378	0.271	-0.153	0.909	0.1631		-0.285	0.286	-0.846	0.275	0.3185		-0.048	0.254	-0.546	0.450	0.8504		0.081	0.231	-0.372	0.534	0.7268	
Conscientiousness	-0.130	0.138	-0.399	0.140	0.3459		0.114	0.123	-0.127	0.356	0.3534		0.206	0.141	-0.070	0.482	0.1432		0.122	0.121	-0.116	0.360	0.3138		0.063	0.109	-0.151	0.276	0.5636	
Extraversion	0.100	0.168	-0.230	0.429	0.5538		0.247	0.166	-0.078	0.572	0.1359		0.528	0.181	0.174	0.882	0.0035	<0.05	0.404	0.158	0.094	0.714	0.0107	<0.05	0.290	0.137	0.021	0.559	0.0349	<0.05
Openness	0.452	0.121	0.214	0.690	0.0002	<0.05	0.270	0.107	0.059	0.480	0.0120	<0.05	0.111	0.123	-0.130	0.352	0.3661		0.263	0.108	0.052	0.475	0.0147	<0.05	0.252	0.092	0.072	0.432	0.0062	<0.05
Neuroticism	-0.012	0.036	-0.083	0.059	0.7432		-0.069	0.036	-0.139	0.001	0.0546		-0.149	0.040	-0.228	-0.071	0.0002	<0.05	-0.111	0.036	-0.181	-0.041	0.0018	<0.05	-0.094	0.032	-0.157	-0.032	0.0030	<0.05
Risk tolerance	0.152	0.039	0.075	0.229	0.0001	<0.05	0.072	0.040	-0.006	0.150	0.0694		0.397	0.043	0.313	0.481	2.77E-20	<0.05	0.290	0.038	0.216	0.364	2.16E-14	<0.05	0.144	0.033	0.080	0.209	1.19E-05	<0.05
Height	0.095	0.030	0.037	0.153	0.0013	<0.05	0.071	0.028	0.016	0.127	0.0122	<0.05	0.123	0.036	0.052	0.194	0.0006	<0.05	0.115	0.030	0.056	0.173	0.0001	<0.05	0.113	0.024	0.066	0.161	2.29E-06	<0.05
Educational achievement	0.401	0.051	0.302	0.501	2.79E-15	<0.05	0.415	0.056	0.305	0.524	1.14E-13	<0.05	0.331	0.056	0.220	0.441	4.22E-09	<0.05	0.415	0.054	0.310	0.520	1.05E-14	<0.05	0.438	0.047	0.345	0.531	2.61E-20	<0.05
Health and Well-Being																														
ADHD	-0.130	0.056	-0.240	-0.020	0.0204	<0.05	-0.168	0.056	-0.278	-0.058	0.0027	<0.05	-0.009	0.058	-0.123	0.105	0.8776		-0.082	0.053	-0.185	0.021	0.1196		-0.178	0.049	-0.274	-0.081	0.0003	<0.05
Alcohol misuse	-0.001	0.046	-0.092	0.090	0.9816		-0.033	0.048	-0.128	0.062	0.4916		0.070	0.054	-0.036	0.175	0.1980		0.027	0.047	-0.066	0.120	0.5696		0.028	0.043	-0.057	0.113	0.5132	
Autism	0.045	0.063	-0.078	0.168	0.4764		0.120	0.057	0.009	0.232	0.0340	<0.05	-0.078	0.065	-0.206	0.049	0.2300		0.014	0.056	-0.096	0.124	0.7985		-0.014	0.050	-0.113	0.085	0.7767	
Schizophrenia	0.263	0.039	0.186	0.340	1.84E-11	<0.05	0.240	0.036	0.170	0.310	1.91E-11	<0.05	0.115	0.043	0.032	0.199	0.0069	<0.05	0.208	0.036	0.139	0.278	4.50E-09	<0.05	0.206	0.032	0.144	0.267	6.73E-11	<0.05
Bipolar disorder	0.281	0.045	0.194	0.369	3.11E-10	<0.05	0.171	0.042	0.088	0.254	5.25E-05	<0.05	0.224	0.045	0.136	0.312	5.49E-07	<0.05	0.244	0.040	0.165	0.323	1.38E-09	<0.05	0.241	0.036	0.171	0.312	1.90E-11	<0.05
BMI	-0.290	0.033	-0.354	-0.226	5.45E-19	<0.05	-0.216	0.032	-0.279	-0.153	2.14E-11	<0.05	-0.068	0.033	-0.133	-0.002	0.0422	<0.05	-0.166	0.031	-0.227	-0.106	6.06E-08	<0.05	-0.163	0.027	-0.217	-0.109	2.81E-09	<0.05
Cancer illness	0.037	0.086	-0.132	0.205	0.6689		0.000101244	0.081	-0.158	0.158	0.9990		0.037	0.100	-0.159	0.234	0.7094		0.048	0.084	-0.117	0.212	0.5720		0.065	0.071	-0.074	0.204	0.3589	
Non-cancer illness	-0.210	0.042	-0.292	-0.128	4.81E-07	<0.05	-0.178	0.042	-0.260	-0.097	1.87E-05	<0.05	-0.115	0.046	-0.205	-0.025	0.0121	<0.05	-0.167	0.041	-0.248	-0.086	5.26E-05	<0.05	-0.192	0.035	-0.261	-0.123	4.94E-08	<0.05
Subjective wellbeing	-0.036	0.057	-0.148	0.077	0.5333		0.057	0.054	-0.049	0.164	0.2933		0.207	0.062	0.086	0.328	0.0008	<0.05	0.129	0.053	0.026	0.233	0.0144	<0.05	0.133	0.047	0.042	0.225	0.0043	<0.05
Longevity	0.130	0.087	-0.040	0.301	0.1333		0.062	0.084	-0.103	0.227	0.4642		-0.099	0.085	-0.266	0.068	0.2463		-0.008	0.078	-0.161	0.145	0.9219		0.062	0.072	-0.078	0.203	0.3860	
Cannabis use	0.350	0.057	0.237	0.462	1.16E-09	<0.05	0.175	0.054	0.069	0.281	0.0012	<0.05	0.181	0.058	0.067	0.295	0.0018	<0.05	0.235	0.054	0.129	0.341	1.36E-05	<0.05	0.216	0.048	0.122	0.311	7.76E-06	<0.05
Nsex	0.129	0.036	0.059	0.199	0.0003	<0.05	-0.045	0.036	-0.116	0.027	0.2200		0.183	0.037	0.110	0.256	9.11E-07	<0.05	0.120	0.034	0.053	0.187	0.0005	<0.05	0.019	0.032	-0.044	0.082	0.5555	
FSEX	0.220	0.039	0.142	0.297	2.62E-08	<0.05	0.263	0.043	0.178	0.348	1.40E-09	<0.05	0.015	0.043	-0.068	0.099	0.7186		0.140	0.038	0.066	0.215	0.0002	<0.05	0.241	0.034	0.173	0.308	3.05E-12	<0.05
Career Success																														
Income	0.299	0.053	0.196	0.403	1.41E-08	<0.05	0.310	0.058	0.197	0.423	7.29E-08	<0.05	0.528	0.065	0.401	0.654	2.94E-16	<0.05	0.462	0.058	0.347	0.577	2.66E-15	<0.05	0.449	0.051	0.349	0.549	1.74E-18	<0.05
Job satisfaction	0.084	0.080	-0.073	0.240	0.2939		0.033	0.075	-0.115	0.180	0.6640		0.196	0.080	0.039	0.353	0.0144	<0.05	0.123	0.073	-0.021	0.266	0.0938		0.069	0.065	-0.059	0.198	0.2903	
Occupational status	0.405	0.066	0.276	0.535	8.03E-10	<0.05	0.504	0.072	0.363	0.645	2.51E-12	<0.05	0.535	0.080	0.379	0.692	2.15E-11	<0.05	0.567	0.074	0.423	0.712	1.53E-14	<0.05	0.543	0.066	0.415	0.672	1.22E-16	<0.05
Number ever born (men)	-0.167	0.055	-0.275	-0.058	0.0027	<0.05	-0.191	0.055	-0.298	-0.083	0.0005	<0.05	0.130	0.062	0.008	0.252	0.0361	<0.05	-0.028	0.054	-0.134	0.079	0.6087		-0.095	0.048	-0.189	-0.002	0.0456	<0.05
Number ever born (women)	-0.153	0.047	-0.245	-0.061	0.0011	<0.05	-0.138	0.046	-0.228	-0.048	0.0026	<0.05	-0.074	0.051	-0.174	0.027	0.1496		-0.123	0.045	-0.211	-0.034	0.0066	<0.05	-0.109	0.039	-0.185	-0.033	0.0050	<0.05
Age first birth	0.313	0.045	0.224	0.401	3.79E-12	<0.05	0.319	0.047	0.227	0.410	9.19E-12	<0.05	0.206	0.048	0.112	0.299	1.63E-05	<0.05	0.290	0.044	0.205	0.376	2.80E-11	<0.05	0.338	0.039	0.262	0.415	5.11E-18	<0.05

Table S15. Comparison of genetic correlation between creativity phenotypes and outcome variables, partialling out intelligence

	Artistic creativity vs. Scientific creativity	Artistic creativity vs. Managerial creativity	Scientific creativity vs. Managerial creativity
**Personal Traits**			
Agreeableness	0.1820	0.7244	0.0923
Conscientiousness	0.1864	0.0880	0.6238
Extraversion	0.5319	0.0826	0.2521
Openness to experience	0.2611	0.0484	0.3309
Neuroticism	0.2648	0.0110	0.1329
Risk tolerance	0.1545	2.71E-05	3.03E-08
Height	0.5587	0.5463	0.2559
Educational achievement	0.8624	0.3497	0.2894
**Health and Well-Being**			
ADHD	0.6328	0.1337	0.0489
Alcohol misuse	0.6309	0.3215	0.1563
Autism	0.3705	0.1743	0.0215
Schizophrenia	0.6647	0.0106	0.0248
Bipolar disorder	0.0730	0.3653	0.3887
BMI	0.1060	1.83E-06	0.0014
No of cancer illness	0.7559	0.9960	0.7721
No of non-cancer illness	0.5907	0.1267	0.3092
Subjective wellbeing	0.2400	0.0041	0.0694
Longevity	0.5699	0.0597	0.1804
Cannabis use	0.0273	0.0397	0.9381
NSEX (Number of sexual partners)	0.0007	0.2981	1.26E-05
FSEX (Age first had sexual intercourse)	0.4613	0.0004	4.85E-05
**Career success**			
Income	0.8891	0.0062	0.0120
Job satisfaction	0.6411	0.3213	0.1372
Occupational status	0.3129	0.2103	0.7707
Number ever born (men)	0.7555	0.0004	0.0001
Number ever born (women)	0.8221	0.2527	0.3476
Age first birth	0.9244	0.1035	0.0909

Table S16. Genetic correlations between creativity and outcome variables, partialling out educational achievement

**Phenotypes**	**Artistic creativity**						**Scientific creativity**						**Managerial creativity**						**Overall occupational creativity**						**Creative achievement**					
			95% CI						95% CI						95% CI						95% CI						95% CI			
	r_g	S.E.	Lower	Upper	*P*	FDR	r_g	S.E.	Lower	Upper	*P*	FDR	r_g	S.E.	Lower	Upper	*P*	FDR	r_g	S.E.	Lower	Upper	*P*	FDR	r_g	S.E.	Lower	Upper	*P*	FDR
Personal Traits																														
Agreeableness	-0.359	0.265	-0.878	0.161	0.176		0.180	0.205	-0.222	0.582	0.380		-0.451	0.264	-0.968	0.067	0.088		-0.255	0.209	-0.665	0.155	0.222		-0.135	0.167	-0.461	0.192	0.420	
Conscientiousness	-0.185	0.131	-0.441	0.071	0.156		0.037	0.101	-0.161	0.235	0.715		0.151	0.130	-0.104	0.406	0.245		0.052	0.103	-0.149	0.254	0.612		-0.014	0.084	-0.178	0.150	0.869	
Extraversion	-0.036	0.158	-0.346	0.274	0.820		0.067	0.134	-0.196	0.330	0.618		0.397	0.169	0.066	0.728	0.019	<0.05	0.239	0.135	-0.026	0.503	0.077		0.110	0.109	-0.103	0.323	0.311	
Openness	0.281	0.109	0.069	0.494	0.010	<0.05	0.103	0.086	-0.065	0.272	0.230		-0.025	0.113	-0.247	0.197	0.827		0.093	0.090	-0.083	0.269	0.301		0.073	0.069	-0.061	0.208	0.286	
Neuroticism	0.066	0.033	0.001	0.130	0.046	<0.05	0.004	0.026	-0.047	0.054	0.889		-0.089	0.033	-0.155	-0.024	0.007	<0.05	-0.036	0.027	-0.088	0.016	0.175		-0.016	0.021	-0.057	0.025	0.443	
Risk tolerance	0.096	0.035	0.028	0.164	0.006	<0.05	-0.008	0.030	-0.067	0.050	0.781		0.340	0.040	0.262	0.419	2.10E-17	<0.05	0.218	0.031	0.157	0.279	3.14E-12	<0.05	0.065	0.024	0.018	0.113	0.007	<0.05
Intelligence	-0.076	0.044	-0.162	0.010	0.083		0.032	0.041	-0.048	0.111	0.432		-0.010	0.051	-0.111	0.090	0.838		-0.010	0.041	-0.090	0.070	0.806		0.004	0.031	-0.056	0.064	0.893	
Height	0.034	0.027	-0.019	0.087	0.209		0.012	0.022	-0.031	0.054	0.593		0.075	0.033	0.011	0.139	0.022	<0.05	0.054	0.024	0.006	0.101	0.027	<0.05	0.050	0.017	0.017	0.083	0.003	<0.05
Health and Well-Being																														
ADHD	0.075	0.052	-0.026	0.176	0.148		0.044	0.039	-0.033	0.120	0.263		0.160	0.052	0.058	0.261	0.002	<0.05	0.130	0.041	0.050	0.209	0.001	<0.05	0.046	0.030	-0.013	0.106	0.128	
Alcohol misuse	-0.035	0.043	-0.119	0.050	0.418		-0.062	0.037	-0.135	0.012	0.099		0.045	0.048	-0.049	0.139	0.350		-0.004	0.038	-0.079	0.071	0.920		-0.003	0.032	-0.066	0.059	0.917	
Autism	-0.002	0.056	-0.112	0.108	0.968		0.091	0.043	0.008	0.175	0.031	<0.05	-0.108	0.058	-0.222	0.007	0.065		-0.022	0.044	-0.108	0.063	0.612		-0.050	0.032	-0.114	0.013	0.121	
Schizophrenia	0.119	0.035	0.051	0.187	0.001	<0.05	0.030	0.028	-0.024	0.084	0.278		-0.033	0.038	-0.107	0.042	0.392		0.021	0.028	-0.035	0.077	0.459		-3.58E-04	0.023	-0.045	0.044	0.987	
Bipolar disorder	0.121	0.042	0.038	0.203	0.004	<0.05	-0.041	0.035	-0.109	0.027	0.238		0.070	0.041	-0.011	0.151	0.091		0.049	0.034	-0.018	0.117	0.152		0.029	0.028	-0.025	0.084	0.290	
BMI	-0.136	0.030	-0.194	-0.078	4.70E-06	<0.05	-0.041	0.026	-0.092	0.010	0.116		0.067	0.031	0.007	0.127	0.029	<0.05	0.003	0.026	-0.047	0.053	0.905		0.018	0.020	-0.021	0.056	0.361	
Cancer illness	0.045	0.079	-0.109	0.199	0.567		-0.006	0.065	-0.134	0.121	0.925		0.037	0.092	-0.143	0.217	0.685		0.047	0.070	-0.090	0.184	0.502		0.062	0.052	-0.040	0.164	0.230	
Non-cancer illness	-0.069	0.038	-0.142	0.005	0.067		-0.022	0.031	-0.084	0.039	0.474		0.006	0.040	-0.072	0.084	0.885		-0.015	0.033	-0.079	0.049	0.644		-0.030	0.025	-0.080	0.019	0.228	
Subjective wellbeing	-0.111	0.054	-0.216	-0.005	0.040	<0.05	-0.037	0.043	-0.123	0.048	0.390		0.137	0.058	0.023	0.251	0.019	<0.05	0.041	0.045	-0.047	0.129	0.358		0.038	0.036	-0.033	0.109	0.293	
Longevity	0.019	0.080	-0.138	0.175	0.817		-0.062	0.066	-0.191	0.067	0.344		-0.195	0.080	-0.351	-0.039	0.014	<0.05	-0.129	0.065	-0.257	-4.10E-4	0.049	<0.05	-0.067	0.053	-0.170	0.037	0.206	
Cannabis Use	0.203	0.050	0.105	0.300	4.60E-05	<0.05	0.019	0.040	-0.060	0.099	0.630		0.059	0.051	-0.041	0.158	0.247		0.081	0.042	-0.001	0.163	0.054		0.053	0.034	-0.014	0.120	0.123	
Nsex	0.165	0.032	0.102	0.228	2.52E-07	<0.05	-0.003	0.028	-0.057	0.052	0.928		0.215	0.034	0.148	0.283	4.16E-10	<0.05	0.160	0.028	0.106	0.215	9.92E-09	<0.05	0.062	0.023	0.017	0.107	0.007	<0.05
FSEX	-0.043	0.027	-0.097	0.010	0.114		-0.010	0.024	-0.058	0.038	0.670		-0.202	0.030	-0.262	-0.143	3.15E-11	<0.05	-0.133	0.024	-0.179	-0.086	2.17E-08	<0.05	-0.048	0.018	-0.084	-0.012	0.009	<0.05
Career Success																														
Income	0.011	0.039	-0.065	0.088	0.767		0.025	0.032	-0.038	0.088	0.435		0.296	0.042	0.215	0.378	1.33E-12	<0.05	0.172	0.034	0.104	0.239	5.98E-07	<0.05	0.144	0.029	0.088	0.201	5.42E-07	<0.05
Job satisfaction	0.087	0.074	-0.058	0.232	0.238		0.040	0.057	-0.071	0.152	0.478		0.201	0.073	0.059	0.343	0.006	<0.05	0.129	0.059	0.012	0.245	0.030	<0.05	0.076	0.047	-0.015	0.168	0.102	
Occupational status	0.038	0.053	-0.065	0.142	0.468		0.106	0.048	0.012	0.200	0.027	<0.05	0.224	0.062	0.103	0.345	3.01E-04	<0.05	0.176	0.052	0.073	0.279	7.88E-04	<0.05	0.128	0.043	0.043	0.213	0.003	<0.05
Number ever born (men)	-0.051	0.051	-0.150	0.048	0.317		-0.077	0.042	-0.159	0.004	0.063		0.223	0.056	0.112	0.333	7.86E-05	<0.05	0.088	0.044	0.002	0.175	0.045	<0.05	0.026	0.034	-0.040	0.092	0.435	
Number ever born (women)	-0.045	0.043	-0.129	0.039	0.292		-0.035	0.036	-0.105	0.035	0.332		0.011	0.047	-0.081	0.104	0.808		-0.016	0.038	-0.090	0.058	0.671		0.003	0.029	-0.054	0.059	0.926	
Age first birth	-0.009	0.040	-0.086	0.069	0.824		-0.022	0.036	-0.092	0.048	0.537		-0.063	0.041	-0.144	0.018	0.127		-0.048	0.034	-0.115	0.020	0.166		-0.019	0.027	-0.073	0.034	0.472	

Table S17. Comparison of genetic correlations between creativity phenotypes and outcome variables, partialling out educational achievement

	Artistic creativity vs. Scientific creativity	Artistic creativity vs. Managerial creativity	Scientific creativity vs. Managerial creativity
**Personal Traits**			
Agreeableness	0.1079	0.8055	0.0591
Conscientiousness	0.1781	0.0679	0.4878
Extraversion	0.6195	0.0611	0.1256
Openness to experience	0.1985	0.0511	0.3682
Neuroticism	0.1369	0.0009	0.0271
Risk tolerance	0.0231	4.19E-06	3.06E-12
Intelligence	0.0707	0.3304	0.5166
Height	0.5173	0.3378	0.1068
**Health and Well-Being**			
ADHD	0.6318	0.2433	0.0729
Alcohol misuse	0.6372	0.2163	0.0800
Autism	0.1839	0.1928	0.0057
Schizophrenia	0.0444	0.0032	0.1837
Bipolar disorder	0.0031	0.3914	0.0401
BMI	0.0159	2.01E-06	0.0073
No of cancer illness	0.6167	0.9489	0.6998
No of non-cancer illness	0.3426	0.1731	0.5782
Subjective wellbeing	0.2906	0.0018	0.0163
Longevity	0.4345	0.0580	0.1990
Cannabis use	0.0042	0.0420	0.5453
NSEX (Number of sexual partners)	7.23E-05	0.2897	8.07E-07
FSEX (Age first had sexual intercourse)	0.3688	0.0001	9.17E-07
**Career success**			
Income	0.7862	5.90E-07	2.77E-07
Job satisfaction	0.6155	0.2737	0.0821
Occupational status	0.3431	0.0225	0.1314
Number ever born (men)	0.6840	0.0003	1.85E-05
Number ever born (women)	0.8537	0.3745	0.4349
Age first birth	0.8041	0.3427	0.4518

Table S18. Constructing PGS for creativity in UKB follow-up cohort, Add Health cohort, and WLS cohort

Creativity Phenotypes	Method	UKB Follow-up						Add Health						WLS					
		P_t cut-off to generate PGS	Pseudo-R2 (PGS)	Pseudo-R2 (cov+PGS)	Coefficient	P-value	# SNP included	P_t cut-off to generate PGS	Pseudo-R2 (PGS)	Pseudo-R2 (cov+PGS)	Coefficient	P-value	# SNP included	P_t cut-off to generate PGS	Pseudo-R2 (PGS)	Pseudo-R2 (cov+PGS)	Coefficient	P-value	# SNP included
Artistic creativity	PRSice	P_t < 0.0450	0.0129	0.05637	0.0510	3.89E-13	21994	P_t < 0.0040	0.0054	0.0529	-0.0563	0.1233	5033	P_t < 5.01e-05	0.0028	0.0763	0.2950	0.1634	52
		P_t < 0.01	0.0074	0.0508	0.0423	4.18E-08	13721	P_t < 0.01	0.0094	0.0568	-0.0440	0.0433	14701	P_t < 0.01	0.0014	0.0749	0.0368	0.3233	4036
		P_t < 1.00E-04	0.0039	0.0473	0.1527	6.53E-05	328	P_t < 1.00E-04	0.0042	0.0516	-0.1532	0.1756	317	P_t < 1.00E-04	0.0003	0.0737	0.0689	0.6725	98
	PRS-CS		0.00569062	0.0491201	3.12266	2.06E-06	153908		0.00042753	0.0478444	-0.323654	0.6647	1109148		0.00267584	0.0761472	1.42982	0.170258	481467
Scientific creativity	PRSice	P_t < 0.0094	0.0108	0.2177	0.0838	7.87E-30	9929	P_t < 5.20E-02	0.0053	0.0985	-0.0474	0.1481	25620	P_t < 0.076	0.0210	0.2440	0.1754	6.72E-05	11510
		P_t < 0.01	0.0130	0.2199	0.0709	1.24E-35	14635	P_t < 0.01	0.0006	0.0938	0.0183	0.6267	15361	P_t < 0.01	0.0073	0.2303	0.1294	0.0180	4466
		P_t < 1.00E-04	0.0036	0.2105	0.1685	4.70E-11	456	P_t < 1.00E-04	0.0004	0.0936	-0.0686	0.6942	434	P_t < 1.00E-04	0.0020	0.2250	0.2655	0.2170	160
	PRS-CS		0.0134156	0.220319	4.57281	1.35E-36	153910		2.34E-05	0.0931831	0.0753833	0.9235	1109135		0.0308134	0.253813	4.86532	1.68E-06	481465
Managerial creativity	PRSice	P_t < 0.0820	0.0043	0.1157	0.0402	2.30E-15	34378	P_t < 1.40E-02	0.0021	0.0525	-0.0430	0.1478	11549	P_t < 7.00E-04	0.0059	0.0816	0.3505	0.0065	425
		P_t < 0.01	0.0042	0.1155	0.0501	5.73E-15	13445	P_t < 0.01	0.0007	0.0511	-0.0197	0.4062	14263	P_t < 0.01	0.0003	0.0760	0.0282	0.5666	3992
		P_t < 1.00E-04	0.0012	0.1125	0.1329	4.01E-05	340	P_t < 1.00E-04	0.0010	0.0515	-0.1291	0.3100	313	P_t < 1.00E-04	0.0019	0.0776	0.3697	0.1192	100
	PRS-CS		0.00198331	0.113336	2.15721	7.55E-08	153903		0.00146935	0.0519048	-0.487875	0.2268	1109169		0.00223	0.0779474	1.21618	0.0939	481465
Overall occupational creativity	PRSice	P_t < 0.0180	0.0077	0.1472	0.0787	4.25E-34	16357	P_t < 2.40E-02	0.0027	0.0573	-0.0449	0.0793	16931	P_t < 5.20E-02	0.0068	0.1184	0.1171	0.0008	9473
		P_t < 0.01	0.0074	0.1469	0.0715	1.15E-32	14958	P_t < 0.01	0.0021	0.0567	-0.0347	0.1202	15953	P_t < 0.01	0.0029	0.1145	0.0891	0.0289	4663
		P_t < 1.00E-04	0.0035	0.1429	0.2187	3.90E-16	474	P_t < 1.00E-04	0.0021	0.0567	-0.1549	0.1233	464	P_t < 1.00E-04	0.0013	0.1129	0.2383	0.1389	165
	PRS-CS		0.00813448	0.147594	3.38063	1.04E-35	153902		1.26E-03	0.0558261	-0.399143	0.2310	1109154		5.95E-03	0.117575	1.54841	0.001	481459
Creative achievement	PRSice	P_t < 0.0710	0.0097	0.0485	0.0789	7.63E-70	39110	P_t < 1.00E-04	0.0005	0.0144	0.0839	0.1623	736	P_t < 1	0.0067	0.0305	0.0903	9.37E-10	35305
		P_t < 0.01	0.0116	0.0504	0.0967	2.54E-83	18140	P_t < 0.01	0.0001	0.0140	0.0102	0.4795	19070	P_t < 0.01	0.0060	0.0298	0.1125	7.53E-09	6093
		P_t < 1.00E-04	0.0064	0.0452	0.2615	1.14E-46	879	P_t < 1.00E-04	0.0002	0.0141	0.0451	0.3993	844	P_t < 1.00E-04	0.0042	0.0279	0.2640	1.45E-06	395
	PRS-CS		0.0103091	0.0490736	1.32483	3.11E-74	153901		1.68E-08	0.0139158	0.0006122	0.997	1109148		0.00980053	0.0335398	0.646353	1.52E-13	481463
