# Supplementary material for: A genome-wide association study of occupational creativity and its relations with well-being and career success
Source: Commun Biol. 2024 Sep 5;7:1092. doi: 10.1038/s42003-024-06686-5 (PMC11377709; doi:10.1038/s42003-024-06686-5)
Supplement: Supplementary file 2 — Reporting summary [file 42003_2024_6686_MOESM2_ESM.pdf]

nature portfolio

Corresponding author(s): Zhaoli Song, Wen-Dong Li, Qiao Fan, Xin Zhang

Last updated by author(s): Jul 27, 2024

Reporting Summary

Nature Portfolio wishes to improve the reproducibility of the work that we publish. This form provides structure for consistency and transparency in reporting. For further information on Nature Portfolio policies, see our Editorial Policies and the Editorial Policy Checklist.

Statistics

For all statistical analyses, confirm that the following items are present in the figure legend, table legend, main text, or Methods section.

n/a	Confirmed	
☐	☒	The exact sample size (*n*) for each experimental group/condition, given as a discrete number and unit of measurement
☐	☒	A statement on whether measurements were taken from distinct samples or whether the same sample was measured repeatedly
☐	☒	The statistical test(s) used AND whether they are one- or two-sided<br>*Only common tests should be described solely by name; describe more complex techniques in the Methods section.*
☐	☒	A description of all covariates tested
☐	☒	A description of any assumptions or corrections, such as tests of normality and adjustment for multiple comparisons
☐	☒	A full description of the statistical parameters including central tendency (e.g. means) or other basic estimates (e.g. regression coefficient) AND variation (e.g. standard deviation) or associated estimates of uncertainty (e.g. confidence intervals)
☐	☒	For null hypothesis testing, the test statistic (e.g. *F*, *t*, *r*) with confidence intervals, effect sizes, degrees of freedom and *P* value noted<br>*Give P values as exact values whenever suitable.*
☒	☐	For Bayesian analysis, information on the choice of priors and Markov chain Monte Carlo settings
☒	☐	For hierarchical and complex designs, identification of the appropriate level for tests and full reporting of outcomes
☐	☒	Estimates of effect sizes (e.g. Cohen's *d*, Pearson's *r*), indicating how they were calculated

Our web collection on statistics for biologists contains articles on many of the points above.

Software and code

Policy information about availability of computer code

Data collection	Each study collected data individually with the method/software described in the method or supplementary file.
Data analysis	GWAS analysis, meta-analysis, and post-GWAS analysis: BOLT-LMM v.2.3.2 (https://data.broadinstitute.org/alkesgroup/BOLT-LMM/downloads) , METAL (the version released on March 2011), GWAMA v2.2.2, FUMA v1.3.6a, ANNOVAR (the version released on 8th June 2020), GCTA 1.92.4 beta version, and LocusZoom v1.4.<br>Heritability and genetic correlation: LDSC v1.0.1<br>Genomic SEM: https://github.com/MichelNivard/GenomicSEM)

For manuscripts utilizing custom algorithms or software that are central to the research but not yet described in published literature, software must be made available to editors and reviewers. We strongly encourage code deposition in a community repository (e.g. GitHub). See the Nature Portfolio guidelines for submitting code & software for further information.

Data

Policy information about availability of data

All manuscripts must include a data availability statement. This statement should provide the following information, where applicable:

- Accession codes, unique identifiers, or web links for publicly available datasets
- A description of any restrictions on data availability
- For clinical datasets or third party data, please ensure that the statement adheres to our policy

The UKB GWAS summary statistics for the five creativity phenotypes will be available on the NHGRI-EPI Catalog of human GWAS https://www.ebi.ac.uk/gwas/. The summary statistics of the top index variants were presented in the article or supporting information, along with the data analyzed in this study.

Research involving human participants, their data, or biological material

Policy information about studies with human participants or human data. See also policy information about sex, gender (identity/presentation), and sexual orientation and race, ethnicity and racism.

Reporting on sex and gender	We used the term of sex in our paper. The findings can apply to both sex. We performed sex based analyses.
Reporting on race, ethnicity, or other socially relevant groupings	We report sex/gender-specific results.
Population characteristics	Individuals of European ancestry in UKB, Add healthy, WLS cohorts
Recruitment	n/a
Ethics oversight	Accessing data resources to conduct this study was approved by the U.K. Biobank (application reference # 37334), Add Health Study (application reference # 11030901), and Wisconsin Longitudinal Study (application reference #98636).

Note that full information on the approval of the study protocol must also be provided in the manuscript.

Field-specific reporting

Please select the one below that is the best fit for your research. If you are not sure, read the appropriate sections before making your selection.

☐ Life sciences ☒ Behavioural & social sciences ☐ Ecological, evolutionary & environmental sciences

For a reference copy of the document with all sections, see nature.com/documents/nr-reporting-summary-flat.pdf

Behavioural & social sciences study design

All studies must disclose on these points even when the disclosure is negative.

Study description	Data are quantitative
Research sample	Dataset source: the U.K. Biobank, Add Health Study and Wisconsin Longitudinal Study.
Sampling strategy	Using existing datasets and using all the available data after merging datasets
Data collection	Using existing datasets
Timing	n/a
Data exclusions	For analyses on creative achievement, Excluded 267936 participants in UK Biobank data analysis because of the merge between genotype and phenotype data. For analyses on occupational creativity, excluded 267936 participants in UK Biobank data analysis because of the merge between genotype and phenotype data. Please see Figure S7-S10 for details.
Non-participation	n/a
Randomization	n/a

Reporting for specific materials, systems and methods

We require information from authors about some types of materials, experimental systems and methods used in many studies. Here, indicate whether each material, system or method listed is relevant to your study. If you are not sure if a list item applies to your research, read the appropriate section before selecting a response.

Materials & experimental systems

n/a	Involved in the study
☒	☐ Antibodies
☒	☐ Eukaryotic cell lines
☒	☐ Palaeontology and archaeology
☒	☐ Animals and other organisms
☒	☐ Clinical data
☒	☐ Dual use research of concern
☒	☐ Plants

Methods

n/a	Involved in the study
☒	☐ ChIP-seq
☒	☐ Flow cytometry
☒	☐ MRI-based neuroimaging

Plants

Seed stocks

Report on the source of all seed stocks or other plant material used. If applicable, state the seed stock centre and catalogue number. If plant specimens were collected from the field, describe the collection location, date and sampling procedures.

Novel plant genotypes

Describe the methods by which all novel plant genotypes were produced. This includes those generated by transgenic approaches, gene editing, chemical/radiation-based mutagenesis and hybridization. For transgenic lines, describe the transformation method, the number of independent lines analyzed and the generation upon which experiments were performed. For gene-edited lines, describe the editor used, the endogenous sequence targeted for editing, the targeting guide RNA sequence (if applicable) and how the editor was applied.

Authentication

Describe any authentication procedures for each seed stock used or novel genotype generated. Describe any experiments used to assess the effect of a mutation and, where applicable, how potential secondary effects (e.g. second site T-DNA insertions, mosiacism, off-target gene editing) were examined.
